# Predicting documented prior intentional self-harm in emergency departments: an interpretable gradient boosting approach using European injury surveillance data

**DOI:** 10.3389/fdgth.2026.1964834

**Published:** 2026-10-14

**Authors:** Marco Giustini, Alessio Pitidis, Cecilia Fazio, Tatiana Alves, Tabea Fian, Huib Valkenberg, Susanne Nijman, Maria Papadakaki, Anna Carannante

**Affiliations:** 1 Department of Environment and Health (DAMSA), Italian National Institute of Health (ISS), Rome, Italy; 2 Epidemiology Department, National Institute of Health Doctor Ricardo Jorge, Lisbon, Portugal; 3 Environmental Health Institute (ISAMB), Faculty of Medicine, University of Lisbon, Lisbon, Portugal; 4 Kuratorium für Verkehrssicherheit, Vienna, Austria; 5 Consumer Safety Institute (VeiligheidNL), Amsterdam, Netherlands; 6 Department of Social Work, School of Health Sciences, Hellenic Mediterranean University, Crete, Greece

**Keywords:** emergency department surveillance, EU-IDB, explainable artificial intelligence, machine learning, missing data, shap, suicide prevention, XGBoost

## Abstract

**Introduction:**

Prior intentional self-harm history is the strongest predictor of completed suicide, yet this information frequently goes uncommunicated during emergency department (ED) presentations. We developed an interpretable gradient boosting model (XGBoost) to predict whether a patient's record in the European Injury Database (EU-IDB) carries a documented prior self-harm flag, using injury characteristics, demographics, and clinical features available at ED attendance.

**Methods:**

The raw dataset comprised 62,467 attendances for intentional self-harm from 18 countries (2008–2023), with 86.2% of outcomes coded Unknown. Formal missingness analysis showed that observed outcome missingness was overwhelmingly explained by country membership (country alone: pseudo-R^2^ = 0.537, AUC = 0.948) and that documentation was positive-biased: across countries, the sparser the documentation, the higher the positivity among documented records (Spearman *ρ* = 0.882). A three-step “Third Way” strategy excluded three countries with structurally absent outcome protocols and defined the target as a documented positive flag vs. its absence, yielding an analytical dataset of 26,554 records (prevalence 13.7%), three times larger than the complete-case alternative.

**Results:**

XGBoost achieved AUC-ROC = 0.800 (95% CI 0.783–0.816), AUC-PR = 0.380, and Brier score = 0.100, significantly outperforming logistic regression (DeLong *p* = 3.93 × 10^−10^). At the optimised threshold (0.080), sensitivity was 80.8%, specificity 62.8%, NPV 95.4%. A time-zero model restricted to variables available at triage achieved AUC-ROC = 0.762 (0.745–0.780) and retained its advantage over the linear baseline (0.729, 0.712–0.746). SHAP analysis identified proximal risk factor documentation, place of occurrence, and patient age as dominant predictors, and a sensitivity analysis excluding the *ProximalRiskFactor* variable (AUC-ROC = 0.765) confirmed robustness to a potentially circular feature. A country prediction test (95.3% accuracy, 14.3× chance) quantified substantial implicit geographic encoding in the clinical features, offering a plausible explanation for the gap between test-set AUC-ROC (0.800), temporal validation (0.674) and group-aware cross-validation (0.658). Country-stratified assessment revealed adequate calibration in most settings but miscalibration in low-volume countries, and fairness analysis identified lower sensitivity in older adults (68.0%; AUC-ROC 0.722, 95% CI 0.617–0.822) as a critical limitation.

**Conclusion:**

This model could serve as a decision support layer within ED workflows, but requires local validation, country-specific recalibration, and embedding within structured post-identification care pathways before deployment.

## 1 Introduction

Across European emergency departments (EDs), self-harm presentations account for a substantial burden of acute care, with between 15% and 25% of patients having a documented history of prior episodes (1, 2), the strongest single predictor of completed suicide (3). In routine ED practice, this critical information is recorded—with varying completeness—in injury surveillance databases such as the European Injury Database (EU-IDB) (4), yet it frequently goes uncommunicated during acute presentations due to patient reluctance to disclose, cross-jurisdictional fragmentation of medical records, and time-pressured triage workflows. The result is a systematic gap between what is documented in the surveillance system and what is available to the treating clinician at the moment of presentation, limiting opportunities for targeted intervention in the highest-risk subgroup (5–7).

Recurrent self-harm is associated with markedly increased risks of completed suicide (1, 2), persistent psychological distress (8), and substantial healthcare utilisation (9). Early identification of individuals with prior episodes during emergency encounters offers a critical window for intervention. Implementation of systematic screening, however, is heterogeneous across care settings: it is well established in psychiatric services and, in several health systems, in primary care, whereas adoption in emergency departments is more uneven, reflecting workload, competing clinical priorities and institutional arrangements. Importantly, evidence from large-scale ED studies demonstrates that while universal screening successfully identifies at-risk individuals, subsequent reductions in suicidal behaviour require structured follow-up interventions beyond screening alone (10). Nevertheless, standardised screening for self-harm history in EDs remains inconsistent, limiting opportunities for prevention and continuity of care (11–13). Recent data from paediatric emergency settings demonstrate that systematic suicide risk screening identifies substantial undetected risk, particularly among patients without psychiatric presenting problems (14).

Traditional risk assessment in EDs relies primarily on clinical judgment, structured interviews, and validated psychometric scales. However, these approaches face several practical limitations. Clinician-administered assessments are time-intensive, require specialised training, and exhibit substantial inter-rater variability (15). Self-report instruments may be affected by patient willingness to disclose sensitive information during acute presentations (16). Moreover, many EDs lack the resources or protocols to systematically evaluate self-harm history for all presentations, resulting in missed opportunities for early intervention (17, 18). Despite decades of clinical research, existing risk stratification tools remain constrained by modest predictive accuracy and poor generalisability beyond their development settings (19, 20).

Machine learning offers a promising avenue for developing data-driven prediction models that can identify patterns in structured electronic health records and injury surveillance databases. Recent studies have demonstrated that gradient boosting and other ensemble methods can achieve strong discrimination in predicting adverse mental health outcomes, including suicide attempts and self-harm recurrence (21–24). However, most published models focus on single-centre datasets or specific national contexts, limiting insights into cross-country generalisability (25). Furthermore, many predictive algorithms operate as “black boxes”, providing risk scores without transparent explanations of the underlying reasoning. The integration of SHAP (SHapley Additive exPlanations) addresses this limitation by quantifying individual feature contributions to predictions (26, 27). Despite these advances, few studies have systematically evaluated model transportability across diverse European healthcare systems or addressed the pervasive challenge of outcome missingness in multinational injury surveillance data (28).

This study aimed to develop and validate an interpretable gradient boosting model that uses routinely collected EU-IDB ED data to predict whether a patient's record carries a documented history of prior intentional self-harm. This is a retrospective pattern-recognition task: the model does not predict future episodes, nor does it infer undocumented clinical history. Rather, it learns which combinations of injury characteristics, demographics, and clinical features -as recorded in the EU-IDB at the time of ED attendance- are associated with a positive prior self-harm flag in the surveillance database. The clinical rationale for this task is that patients whose observable ED patterns are strongly consistent with documented prior history of self-harm represent a subgroup in which a targeted inquiry about previous episodes is particularly likely to be informative. By surfacing these cases automatically from routinely collected data, the model could reduce the burden on clinical staff and decrease the probability of missed opportunities for intervention.

Our specific objectives were fourfold: (1) to formally characterise the missingness mechanism of the primary outcome variable and construct a methodologically defensible analytical dataset through a principled three-step strategy; (2) to build a high-performing classifier benchmarked against a logistic regression baseline, while explicitly preventing geographical data leakage and removing temporally confounded features; (3) to quantify feature importance using SHAP values to enhance model interpretability and assess potential algorithmic bias across demographic subgroups; and (4) to rigorously assess cross-country generalisability through group-aware cross-validation, providing insights into model transportability across diverse European healthcare contexts.

## 2 Materials and methods

### 2.1 Data and study population

This retrospective model development and validation study used cross-sectional registry data from the EU-IDB, a multinational emergency department surveillance system coordinating injury data collection across European countries. In the EU-IDB coding scheme the variable *Intent* records the role of human purpose in the injury event, and code 2 denotes intentional self-harm; records coded Intent = 2 trigger the Intentional Self-Harm Module, which contains the outcome variable used here. The study population therefore consists exclusively of ED attendances for intentional self-harm. The raw dataset comprised 62,467 attendance records from ED attendances across 18 countries, spanning the period 2008–2023. Figure 1 documents the construction of the analytical datasets from the source records.

**Figure 1:**
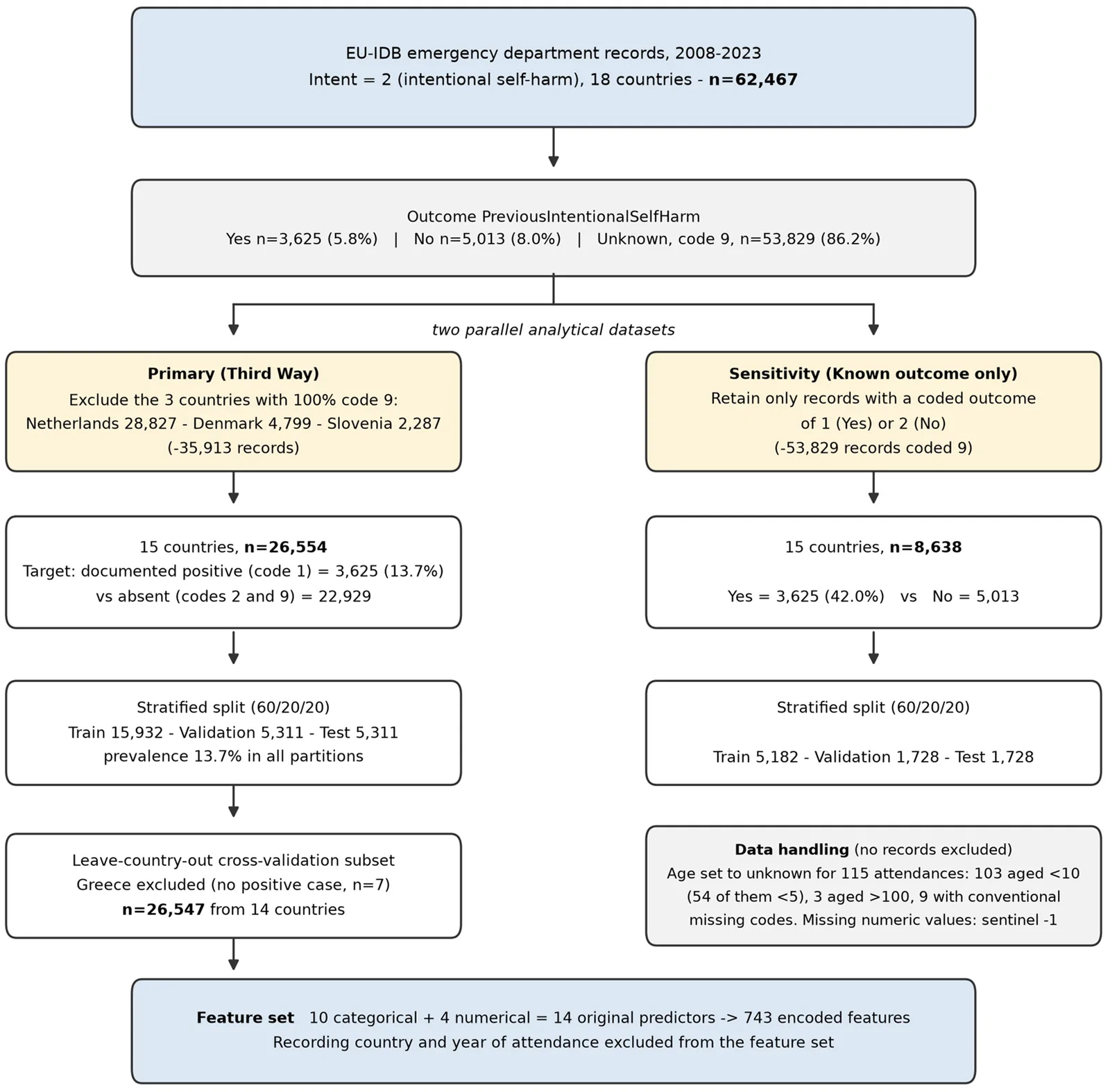
Record flow from the source EU-IDB dataset to the analytical datasets. All records are emergency department attendances coded Intent = 2 (intentional self-harm), for which the Intentional Self-Harm Module containing the outcome variable is completed. Two datasets were constructed in parallel: the primary dataset, excluding the three countries with no coded outcome, and the sensitivity dataset, restricted to records with a coded outcome. No records were excluded on grounds of implausible age.

This inclusion criterion defines the clinical question. The task is not to detect self-harm among undifferentiated ED attendances—these patients have already presented with a self-harm injury and are being treated as such—but to distinguish, among them, those with a previously documented episode from those presenting for the first time. This corresponds to the stated purpose of the outcome variable in the EU-IDB manual, which is to provide insight into the ratio of first-time to repeat attempters, and it is clinically consequential: previous self-harm is the strongest single predictor of subsequent repetition and of completed suicide (1, 2).

The primary outcome was the EU-IDB *PreviousIntentionalSelfHarm* variable, which takes three coded values (1 = Yes, 2 = No, 9 = Unknown); code 9 is an explicit category of the EU-IDB coding scheme, indicating that the information was not available to the coder at registration, rather than an empty or untransmitted field. The modelling target was defined as a documented positive flag (code 1) vs. its absence (codes 2 and 9), so that the outcome is observed for every record.

This variable captures documented history within the scope of information available at ED attendence; it does not represent a verified longitudinal clinical record, and its completeness varies across jurisdictions depending on triage protocols, patient disclosure behaviour, and access to prior medical records. The modelling task was therefore defined as predicting the probability that this documented flag is positive, given the injury and clinical characteristics of the ED attendance. This is explicitly a retrospective identification task: the model learns patterns associated with documented prior history at presentation, and its intended use is to prompt a targeted question about previous episodes in patients already being treated for self-harm, not to detect history that has not been disclosed or recorded.

#### 2.1.1 Outcome missingness characterisation and analytical dataset construction

Inspection of the outcome variable revealed a three-category distribution: Yes (*n* = 3,625, 5.8%), No (*n* = 5,013, 8.0%), and Unknown (*n* = 53,829, 86.2%). Given the extent of missing outcome data, we conducted a formal missingness analysis prior to any exclusion, addressing two questions: (1) what drives missingness, and (2) what are the implications of different assumptions about the true status of Unknown records?

To characterise the missingness mechanism, we fitted three logistic regression models predicting Unknown vs. Known outcome status, using country membership alone (Model A), clinical and demographic variables alone (year of attendance, sex, age group, and proximal risk factor) without country (Model B), and the full combination (Model C). McFadden's pseudo-R^2^ and AUC-ROC were computed for each model. Country alone explained 91.1% of the full-model pseudo-R^2^ (Model A: pseudo-R^2^ = 0.537, AUC = 0.948; Model C: pseudo-R^2^ = 0.589, AUC = 0.963), indicating that observed missingness was overwhelmingly explained by country membership. Stratification by country confirmed that three countries, Netherlands (*n* = 28,827), Denmark (*n* = 4,799), and Slovenia (*n* = 2,287), had 100% Unknown outcome status, collectively accounting for 57.5% of all records. These countries have no outcome collection protocol within the EU-IDB framework and cannot contribute outcome information under any missingness assumption.

To quantify the potential impact of misclassification, we conducted a sensitivity analysis under missing-not-at-random (MNAR) assumptions, computing the implied overall prevalence of history-positive cases if 5%, 10%, 20%, 30%, or 50% of Unknown records were true positives (Supplementary Table 1). Under a 10% MNAR scenario the overall prevalence would rise to 14.4%; under 20%, to 23.0%. These scenarios bound the plausible impact of outcome misclassification but do not identify the true prevalence, which remains unknowable from the observed data.

Based on these findings, the analytical dataset was constructed using a three-step strategy (hereafter referred to as the Third Way) designed to balance sample size against missingness-induced bias:

1. Records from the three countries with 100% outcome missingness (Netherlands, Denmark, Slovenia) were excluded, as they contribute no outcome information under any plausible assumption.
2. Among the remaining 15 countries, records with a Known outcome (Yes or No) were retained as-is.
3. For the purposes of this analysis the outcome data were classified in two groups: (a) positive outcome; (b) negative or unknown outcome. In these selected countries an outcome collection protocol exists, and positive history—when ascertained—is plausibly recorded with priority. This classification is adopted on conservative grounds and stress-tested in the sensitivity analyses.

The resulting analytical dataset comprised 26,554 records from 15 countries, with 3,625 history-positive cases (13.7%) and 22,929 history-negative cases (86.3%), yielding an imbalance ratio of 6.3:1. A sensitivity analysis dataset comprising only records with Known outcome (Yes/No; *n* = 8,638, prevalence 42.0%) was retained for comparison. The recording country of each case was retained solely for sample weighting and cross-validation grouping and was explicitly excluded from the model feature set to prevent geographical data leakage.

### 2.2 Feature engineering

Three EU-IDB variables coded object or substance involvement across three hierarchical layers. We derived two synthetic variables: *ObjectInvolved*, classifying the pattern of populated layers across twelve mutually exclusive integer-coded categories, and *MainObject*, selecting the single most informative object code following a priority rule (direct > underlying > intermediate). A binary COVID-19 period indicator was derived from the year of attendance (1 if year ≥2020, 0 otherwise). Year of attendance was explicitly excluded from the feature set, as it conflates epidemiological trends with temporal variation in documentation completeness. The SHAP analysis from a preliminary model including Year confirmed that its importance shifted sign in 2016, coinciding with changes in national data collection protocols rather than epidemiological events, rendering it uninterpretable as a clinical predictor (Supplementary Table 2).

Systematic data cleaning was applied to all variables. Numeric variables were screened for conventional missing codes and recoded as missing and categorical variables with missing or non-informative codes were consolidated under an “Unknown” category. Age values outside 10–100 years were treated as unknown rather than excluded. This bound was a pragmatic data-cleaning parameter rather than an eligibility criterion and is supported *post hoc* by the age distribution: counts below age 10 are flat and low, in contrast to the steep gradient beginning at age 11, and 61 of these 115 records are below age five, for which the EU-IDB manual directs coders to classify self-harm as unintentional. Five records had recorded ages of 101–113 years. No records were excluded: the age of these 120 attendances (0.2%) was set to be unknown and handled through the sentinel encoding described below.

Missing values in numerical predictors were encoded with a sentinel value (−1) rather than imputed, allowing the gradient boosting algorithm to treat missingness as an informative category. This affected 0.8% of age values and 52.7% of records for number of days in hospital. We interpret the latter as most likely reflecting non-admission but note that this is an operational assumption rather than an established property of the field; the variable is retained only in the full-encounter model and does not enter the time-zero configuration described in Section 2.5.4.

### 2.3 Feature set

The model feature set comprised ten categorical variables (sex of patient, mechanism of injury, main object involved, activity when injured, place of occurrence, time of attendance, proximal risk factor, treatment and follow-up, type of injury, and part of the body injured) and four numerical variables: age of patient, number of days in hospital, COVID-19 period indicator, and the object involvement pattern (*ObjectInvolved*, entered in the model matrix as a single integer-coded variable rather than one-hot encoded). Year of attendance and country were excluded from the feature set.

### 2.4 Data partitioning

The analytical dataset was partitioned by stratified random sampling training (60%), validation (20%), and test (20%) subsets, preserving the 13.7% prevalence across all partitions. To mitigate over-representation of high-volume countries, each training observation received a weight inversely proportional to its country's sample size, normalised to a mean of 1.0 and clipped to [0.5, 5.0]. In practice the clipping is binding for thirteen of the fifteen countries, so the weighting does not rebalance large against small contributors: its net effect is to amplify the three countries contributing fewer than ten training records each (Supplementary Table S3).

### 2.5 Model development

#### 2.5.1 Baseline model: logistic regression

An L1-regularised logistic regression (LASSO) was fitted as a baseline comparator using the identical feature matrix, class weighting, and calibration procedure as the primary model—one-hot encoded categorical variables plus unstandardised numerical variables, including the integer-coded *ObjectInvolved*. Holding the input representation constant isolates algorithmic differences between the two models, at the cost of a representation more naturally suited to tree-based methods than to a linear model. Class imbalance was addressed by setting class weight = 'balanced' (ratio 6.33:1). Regularisation parameter C = 1.0, SAGA solver, maximum 5,000 iterations. We additionally fitted a re-specified linear model addressing four aspects of this specification. *ObjectInvolved* was one-hot encoded rather than entered as an integer; continuous predictors were standardised; the effect of patient age was modelled with a cubic spline basis (five knots) to allow non-linearity; and the regularisation strength was selected by 5-fold cross-validation over ten candidate values of C, optimising average precision, rather than fixed at C = 1. The resulting design matrix comprises 759 columns. This model is reported alongside the baseline described above as a fairness check on the comparison, not as a replacement for it: the shared-representation baseline remains the comparator that isolates algorithmic differences.

#### 2.5.2 Primary model: gradient boosting (XGBoost)

A Gradient Boosting classifier (XGBoost) was implemented with decision trees of maximum depth 6, moderate regularisation (L2 = 1.0, learning rate = 0.07), and stochastic components (subsample = 0.8) to improve generalisation. The scale_pos_weight parameter was set to 6.33 and base_score initialised to the training prevalence (0.137). Training proceeded for up to 2,500 iterations with early stopping after 150 rounds without improvement on validation AUC-PR. The best model was identified at iteration 212.

#### 2.5.3 Probability calibration and threshold optimisation

Isotonic regression calibration was applied *post-hoc* to the predicted probabilities of both models on validation data. The binary decision threshold was optimised using three criteria: Youden's J statistic, F2-score maximisation (*β* = 2), and a 90% sensitivity target. The F2-maximising threshold (0.080) was adopted as the analytical operating point rather than as a clinically optimal cut-off. The choice of *β* = 2 reflects the asymmetric cost structure of this screening context: in preventive screening for self-harm history, the clinical cost of a false negative (i.e., a high-risk patient not identified and therefore not referred for targeted follow-up) substantially exceeds the cost of a false positive (i.e., an unnecessary psychiatric consultation). Setting *β* = 2 reflects the standard rationale of clinical screening contexts, in which recall is prioritised over precision (20). Importantly, the operating threshold of 0.080 was optimised on the aggregated multinational dataset (prevalence = 13.7%) and may require country-specific recalibration in deployment settings with substantially different outcome prevalences, for example, the 3.0% prevalence in Sweden or the 41.5% prevalence in Spain (Supplementary Table S4) would shift the optimal operating point considerably.

#### 2.5.4 Time-zero configuration

Predictors differ in when they become available during an emergency department episode. We classified them into three groups. Available at presentation: sex, age, time of attendance, mechanism of injury, place of occurrence, activity when injured, main object involved, object involvement pattern, type of injury, part of the body injured, and the COVID-19 period indicator. Generated during clinical assessment: *ProximalRiskFactor*. Generated by management decisions or after discharge: *TreatmentAndFollowUp* and *NumberOfDaysInHospital*. Type of injury and body part injured are registered as presentation characteristics in the EU-IDB scheme but may in practice be finalised after initial examination; we retained them in the first group because they describe the presenting injury rather than the clinical assessment.

Two configurations were therefore fitted with otherwise identical procedures. The full-encounter model uses all fourteen predictors and characterises the information accumulated across the whole episode, including documentation practice; it is the configuration on which the analyses reported below are based, because the object of this study is the structure of documented information rather than a deployable tool. The time-zero model is restricted to the eleven predictors available at presentation and represents the only configuration that could operate in real time, immediately after triage registration and before formal psychosocial assessment. Results for both are reported, and the distinction is maintained throughout: performance of the full-encounter model should not be read as an estimate of what a real-time implementation would achieve.

### 2.6 Model evaluation

Model performance was assessed on the held-out test set. Discrimination was quantified through AUC-ROC and AUC-PR. Calibration quality was evaluated via Brier score. At the optimised threshold, we report sensitivity, specificity, precision (PPV), NPV, balanced accuracy, F1, F2, Cohen's *κ*, MCC, and number needed to screen (NNS). The AUC-ROC difference between XGBoost and logistic regression was formally tested using the DeLong method. Net clinical benefits were assessed via Decision Curve Analysis.

### 2.7 Feature interpretability

SHAP values were derived from TreeSHAP on the uncalibrated XGBoost model, applied to the full test set (*n* = 5,311). Global feature importance was computed as mean absolute SHAP value per feature. Age-specific contributions were characterised by aggregating SHAP values across predefined age bands (10–17, 18–24, 25–34, 35–44, 45–54, 55–64, ≥65 years). COVID-19 period SHAP contributions were compared between pre- and post-2020 presentations using Mann–Whitney U test and Cliff's delta.

### 2.8 Fairness analysis

Model performance was stratified by sex and age group, reporting AUC-ROC, sensitivity, specificity, PPV, NPV, false positive rate, and false negative rate for each subgroup.

### 2.9 Generalisability assessment

Group-aware 5-fold cross-validation was performed using GroupKFold with country as the grouping variable, across 14 countries with at least one history-positive case. A sensitivity analysis excluding Sweden, which contributes 96.9% Unknown or No records and yielded near-chance AUC-PR in its validation fold, assessed transportability among countries with more genuine outcome documentation. A further sensitivity analysis repeated the full modelling pipeline on the Known-outcome-only dataset (*n* = 8,638) to assess whether the Third Way classification materially altered feature importance rankings.

A provider identifier is present in the EU-IDB record structure and is populated for 86.5% of the analytical dataset, but it was not used as a grouping variable: its coverage is structurally uneven, being absent for two countries, restricted to the conventional “unknown” code in a third, and ranging from one to over thirty contributing centres per country. Grouping by centre would therefore have removed whole national contributions from the design. The record structure contains no patient identifier, so records represent attendances rather than individuals and repeated attendances by the same person cannot be linked; we could not ensure that all attendances of a given patient fell within the same partition.

To assess transfer across time rather than across countries, we additionally trained the model on attendances recorded before 2020 and tested it on those from 2020 onwards. The COVID-19 period indicator was necessarily excluded from this analysis, being constant within the training period, so the temporal model operates with one predictor fewer than the primary configuration.

### 2.10 Sensitivity analysis: feature circularity assessment

To assess whether the model's discrimination depended critically on a potentially circular feature, we repeated the full modelling pipeline after excluding *ProximalRiskFactor* from the feature set. *ProximalRiskFactor* is documented during the same ED encounter that generates the outcome variable, creating a risk that both variables reflect overlapping clinical judgment. The reduced model was trained, calibrated, and evaluated on the same data partitions as the primary model, and SHAP feature rankings were compared to assess stability.

### 2.11 Country prediction test

To quantify the degree to which clinical features implicitly encode geographic information, providing a mechanistic explanation for the gap between test-set and cross-validation performance, a multiclass XGBoost classifier was trained to predict the recording country from the same feature set used in the primary model. Classification accuracy was compared against chance level (1/k, where k is the number of countries), and XGBoost feature importance (gain) was used to identify which clinical variables carried the most geographic signal.

### 2.12 Country-specific calibration assessment

Calibration quality was assessed within individual countries by computing country-stratified calibration curves using quantile-based binning (3–10 bins depending on sample size) and the mean absolute calibration error (MACE) for each country with at least 50 test-set observations. MACE thresholds of ≤0.05 (well-calibrated) and >0.10 (substantially mis- calibrated) were adopted as pragmatic descriptive benchmarks, consistent with conventional calibration assessment frameworks (29).

### 2.13 Software

All analyses were conducted in Python 3.14, using scikit-learn 1.9.0 for preprocessing, metrics, and calibration; XGBoost 3.4.0 for model training; SHAP 0.52.0 for interpretability; and pandas 3.0.3, NumPy 2.4.6, SciPy 1.18.0, Matplotlib, and Seaborn for data handling and visualisation. All random operations were seeded at 42, and predictions were explicitly restricted to the boosting rounds selected by early stopping.

### 2.14 Ethics and data governance

This study is a secondary analysis of pre-existing records held in the European Injury Database. It involved no prospective recruitment, no contact with patients, no intervention and no collection of new data. The EU-IDB collects anonymised injury surveillance data excluding direct identifiers. The records available to the investigators contain no identifier permitting linkage of attendances to individuals or across attendances, and no re-identification key was accessible to them.

Within the EU-IDB network, EuroSafe acts as Data Controller on behalf of the network partners and the Italian National Institute of Health as Data Processor, providing hosting, data management and analytical services under the Controller's instructions; the national data providers are independent Data Controllers for their respective datasets. Processing is governed by the principles of Article 5 GDPR, including data minimisation, and by technical and organisational security measures pursuant to Article 32, as set out in the agreement governing the hosting of the database and in the EU-IDB Operating Manual. Formal ethics committee review and individual informed consent were not required for the secondary analysis of these records.

## 3 Results

### 3.1 Dataset characteristics and missingness analysis

The raw EU-IDB dataset comprised 62,467 records from 18 countries spanning 2008–2023. Inspection of the primary outcome variable revealed that 53,829 records (86.2%) had unknown outcome status, with only 3,625 (5.8%) documented as history-positive and 5,013 (8.0%) as history-negative. Missingness was predominantly structural and country-level: three countries, Netherlands (*n* = 28,827, 46.1% of the total dataset), Denmark (*n* = 4,799, 7.7%), and Slovenia (*n* = 2,287, 3.7%), recorded no outcome data whatsoever, collectively accounting for 57.5% of all records (Supplementary Table S4). Among the remaining 15 countries, missingness ranged from 9.5% (Romania) to 96.9% (Sweden).

Logistic regression showed that observed outcome missingness was overwhelmingly explained by country membership, which alone achieved pseudo-R^2^ = 0.537 and AUC = 0.948 in predicting Unknown vs. Known status, explaining 91.1% of the full-model pseudo-R^2^ (Supplementary Table S5). Missingness showed a temporal gradient, declining from 97.7% in 2008 to 67.9% in 2016 before stabilising at 80%–87% through 2023, reflecting progressive changes in national data collection protocols. Under a 10% MNAR assumption, the implied overall prevalence would rise to 14.4% (Supplementary Table S1). Documentation completeness and outcome positivity were also strongly related at country level: across the eleven countries contributing at least 100 records with a coded outcome, the proportion of attendances left uncoded correlated with the proportion of positive cases among those coded (Spearman *ρ* = 0.882, *p* = 0.0003; Pearson r = 0.834, *p* = 0.0014). Sweden, where the field is uncoded in 96.9% of attendances, records a positive history in 97.1% of the cases where it is completed; Romania, with 9.5% uncoded, records 32.1%. The relationship holds across three orders of magnitude of national sample size (Supplementary Table S4).

Following the Third Way strategy, the analytical dataset comprised 26,554 records from 15 countries, with 3,625 history-positive cases (13.7%) and 22,929 history-negative cases (86.3%), yielding an imbalance ratio of 6.3:1. Records spanned 2008–2023, with 4,098 records (15.4%) from the COVID-19 period (≥2020).

### 3.2 Cohort description

History-positive cases were more frequently female (55.3% vs. 51.1%) and showed a markedly different proximal risk factor profile: psychological or psychiatric conditions were documented in 44.9% of history-positive cases vs. 19.5% of history-negative cases (SMD = 0.564), while unspecified proximal risk factor predominated among history-negative cases (58.5% vs. 27.2%, SMD = −0.666). Place of occurrence also differed substantially: history-positive cases presented less frequently from private homes (21.0% vs. 38.3%, SMD = −0.385) and more frequently from unspecified residential locations (22.3% vs. 15.6%, SMD = 0.174). Leisure activity was substantially more common among history-negative cases (14.0% vs. 2.7%, SMD = −0.415).

### 3.3 Effect of removing year from the feature set

To quantify the contribution of Year of attendance, a feature that conflates epidemiological trends with documentation completeness shifts, we compared model performance with and without this variable. Removing Year reduced AUC-PR by 2.2 percentage points (0.403 → 0.380) and AUC-ROC by 1.0 points (0.810 → 0.800) on the test set, confirming a modest but real contribution. Critically, cross-validation AUC-ROC improved from 0.646 to 0.658, and fold-to-fold variability decreased (SD 0.030 → 0.027), demonstrating that Year contributed country-specific overfitting rather than genuinely transportable signal. All subsequent results refer to the model without Year.

### 3.4 Model training

One-hot encoding yielded 739 binary features with 98.6% zero values. Combined with four numerical features, the final feature matrix comprised 743 variables. XGBoost training with early stopping identified the best model at iteration 212 (validation AUC-PR 0.381 e AUC-ROC 0.803); baseline validation AUC-PR 0.317 e AUC-ROC 0.768. The logistic regression baseline achieved validation AUC-PR = 0.298 and AUC-ROC = 0.746.

### 3.5 Threshold selection and test-set performance

Threshold optimisation on calibrated validation probabilities produced a Youden's J threshold of 0.130, an F2-maximising threshold of 0.080, and a 90%-sensitivity threshold of 0.060. The F2-maximising threshold (0.080) was adopted as the primary operating point for both models.

On the independent test set (*n* = 5,311), the calibrated XGBoost model achieved AUC-ROC = 0.800 (95% CI 0.783–0.816) and AUC-PR = 0.380 (0.351–0.415), a 2.8-fold improvement over baseline prevalence, with Brier score = 0.100 (Figure 2).

**Figure 2:**
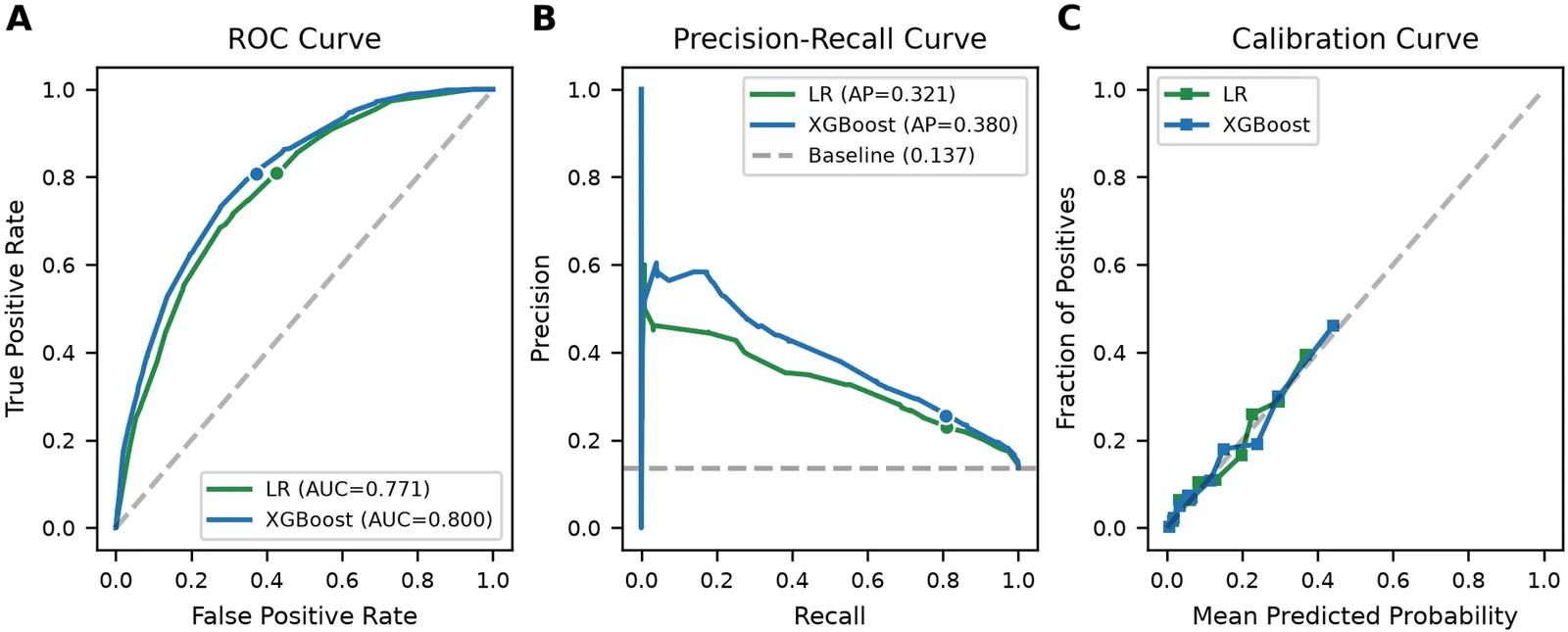
Discrimination and calibration performance of XGBoost and logistic regression on the independent test set (*n* = 5,311). **(A)** Receiver operating characteristic curves; **(B)** precision-recall curves, with the dashed line indicating baseline prevalence (0.137); **(C)** calibration curves after isotonic regression. Points mark each model's F2-maximising operating point.

The logistic regression baseline achieved AUC-ROC = 0.771 (0.755–0.788) and AUC-PR = 0.321 (0.297–0.349). The difference in AUC-ROC was statistically significant (DeLong test: Z = 6.257, *p* = 3.93 × 10⁻^10^). A re-specified linear model, in which *ObjectInvolved* was one-hot encoded, continuous predictors standardised, patient age modelled with a cubic spline basis and the regularisation strength selected by cross-validation, performed essentially identically to this baseline (AUC-ROC = 0.771, 95% CI 0.754–0.789; AUC-PR = 0.326, 0.302–0.355), and the comparison with XGBoost remained significant (DeLong Z = 6.34, *p* = 2.3 × 10⁻^10^) Table 1 reports full operating characteristics across thresholds from 0.05 to 0.90.

**Table 1:** XGBoost operating characteristics across decision thresholds on the independent test set (*n* = 5,311).

Threshold	Sensitivity	Specificity	PPV	NPV	NNS	FP	FN	TP	TN
0.05	0.948	0.380	0.195	0.979	5.14	2,843	38	687	1,743
0.08*	0.808	0.628	0.256	0.954	3.91	1,707	139	586	2,879
0.10	0.808	0.628	0.256	0.954	3.91	1,706	139	586	2,880
0.15	0.730	0.724	0.294	0.944	3.40	1,268	196	529	3,318
0.20	0.650	0.780	0.319	0.934	3.14	1,007	254	471	3,579
0.25	0.623	0.804	0.335	0.931	2.99	899	273	452	3,687
0.30	0.390	0.917	0.427	0.905	2.34	379	442	283	4,207
0.40	0.174	0.980	0.581	0.882	1.72	91	599	126	4,495
0.50	0.044	0.995	0.582	0.868	1.72	23	693	32	4,563
0.60	0.043	0.995	0.574	0.868	1.74	23	694	31	4,563
0.70	0.043	0.995	0.574	0.868	1.74	23	694	31	4,563
0.80	0.000	1.000	—	0.863	—	0	725	0	4,586
0.90	0.000	1.000	—	0.863	—	0	725	0	4,586

*The row marked with an asterisk indicates the selected F2-maximising operating point (0.080). Thresholds between 0.080 and 0.100 yield near-identical classifications because isotonic calibration produces a step function with a plateau across this range. PPV and NNS are undefined at thresholds ≥ 0.80, where no positive predictions are issued.

PPV, positive predictive value; NPV, negative predictive value; NNS, number needed to screen; FP, false positives; FN, false negatives; TP, true positives; TN, true negatives.

At the optimised threshold of 0.080, XGBoost performance metrics were: sensitivity = 80.8%, specificity = 62.8%, precision (PPV) = 25.6%, NPV = 95.4%, balanced accuracy = 71.8%, F2 = 0.564, Cohen's *κ* = 0.228, MCC = 0.302, and number needed to screen (NNS) = 3.9. Table 2 presents the head-to-head model comparison.

**Table 2:** Head-to-head comparison of XGBoost and L1-regularised logistic regression on the independent test set (*n* = 5,311; prevalence = 13.7%).

Metric	Logistic regression	XGBoost
Optimised threshold	0.095	0.080
AUC-ROC	0.771	0.800
AUC-PR	0.321	0.380
Brier score	0.105	0.100
Sensitivity	0.810	0.808
Specificity	0.573	0.628
Precision (PPV)	0.231	0.256
NPV	0.950	0.954
Balanced accuracy	0.691	0.718
Overall accuracy	0.605	0.652
F1	0.359	0.388
F2	0.539	0.564
Cohen's *κ*	0.186	0.228
MCC	0.263	0.302
NNS	4.33	3.91
True positives (TP)	587	586
True negatives (TN)	2,628	2,879
False positives (FP)	1,958	1,707
False negatives (FN)	138	139

Both models used the same feature matrix, class weighting, and isotonic calibration. Thresholds were independently optimised to maximise the F2 score. Highlighted rows indicate primary discrimination metrics.

The time-zero configuration, restricted to the eleven predictors available at presentation (Section 2.5.4), achieved AUC-ROC = 0.762 (95% CI 0.745–0.780) and AUC-PR = 0.321 (0.295–0.350), with an F2-maximising threshold of 0.110. The reduction relative to the full-encounter model was statistically significant (DeLong Z = 6.80, *p* = 1.0 × 10^−11^). At its operating point, sensitivity was 74.8%, specificity 63.3%, precision 24.3% and NNS 4.1. The advantage over the linear baseline was preserved at equal information: restricted to the same eleven predictors, the baseline achieved AUC-ROC = 0.729 (0.712–0.746) and AUC-PR = 0.266 (0.246–0.289), a difference that remained significant (DeLong Z = 5.97, *p* = 2.4 × 10^−9^).

### 3.6 Decision curve analysis

Decision Curve Analysis demonstrated that XGBoost provided positive net clinical benefit over treat-all and treat-none strategies across a clinically relevant range of threshold probabilities (Supplementary Figure S1) and consistently outperformed the logistic regression baseline. The advantage was most pronounced at threshold probabilities between 0.10 and 0.30.

### 3.7 Feature importance

SHAP analysis identified proximal risk factor documentation, age, place of occurrence, and injury-specific characteristics as the features contributing most to predicted prior self-harm history (Figure 3; Supplementary Table S6). The dominant predictor was *ProximalRiskFactor*: Unspecified (mean |SHAP| = 0.465), followed by *ProximalRiskFactor*: Psychological/psychiatric condition (0.230), *PlaceOfOccurrence*: Private home (0.208), and *ActivityWhenInjured*: Leisure (0.194). *ObjectInvolved* (0.140) and *AgeOfPatient* (0.128) also contributed meaningfully.

**Figure 3:**
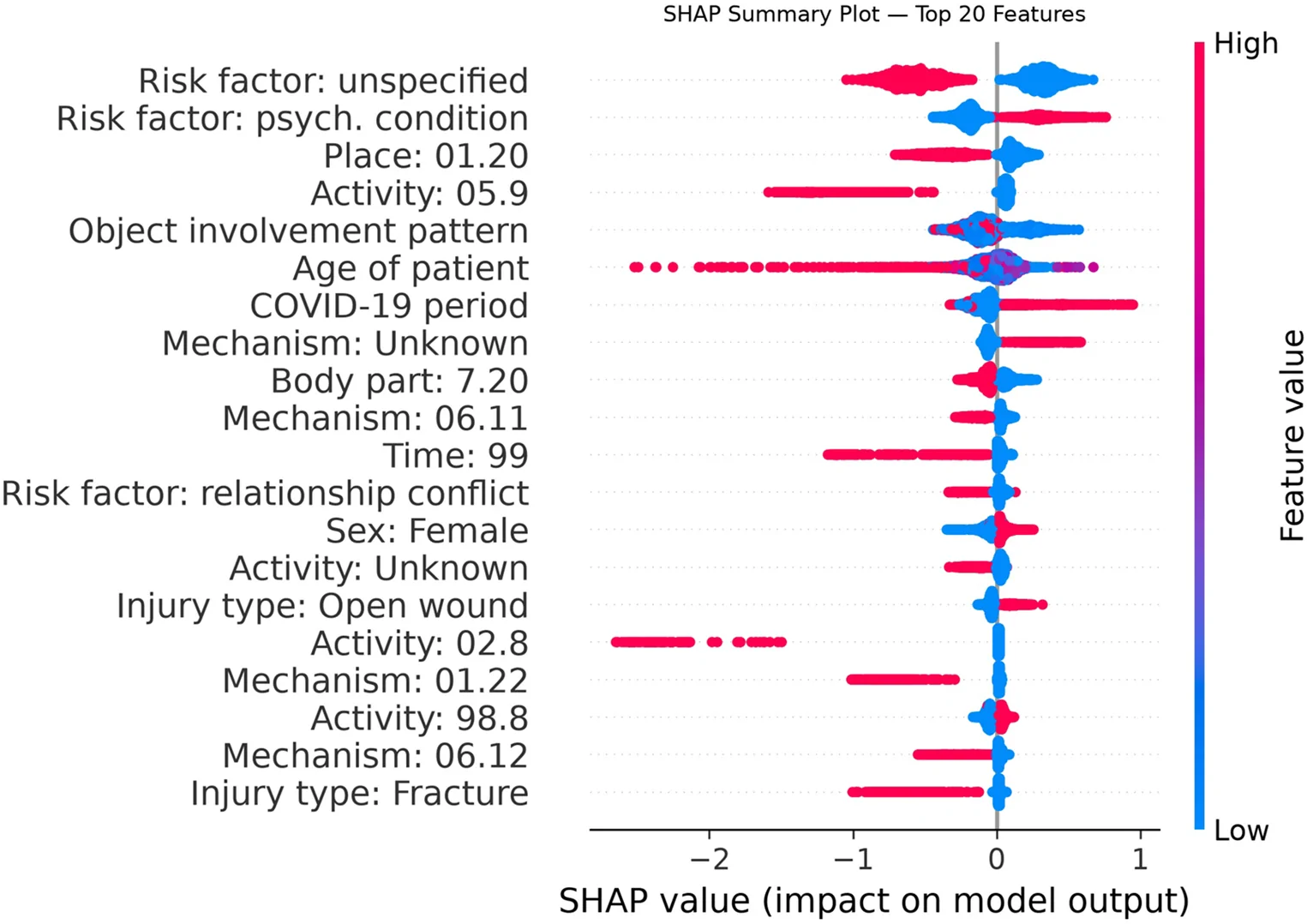
SHAP (SHapley additive exPlanations) summary plot showing the top 20 features by mean absolute SHAP value. Each point represents a single observation in the test set; horizontal position indicates the feature's contribution to the predicted log-odds of documented prior self-harm history (positive = increased predicted probability, negative = decreased). Colour encodes the feature value (red = high, blue = low). Feature names are abbreviated for display; full variable names are given in Supplementary Table 6.

Importantly, the prominence of *ProximalRiskFactor: Unspecified* as the top predictor requires careful interpretation. In the sensitivity analysis on Known-outcome records only, this feature does not appear among the top 10 predictors, strongly suggesting that its dominance in the primary model reflects documentation patterns associated with the Unknown or No classification rather than clinical signal. Readers should interpret this feature's SHAP contribution accordingly. By contrast, *ProximalRiskFactor: Psychological/psychiatric condition* ranks consistently in both models and its association with self-harm repetition is well established in the clinical literature (8, 30). We report this stability as an observation about which explanations survive a change in outcome definition rather than as evidence of clinical determination. COVID-19 period ranked seventh (mean |SHAP| = 0.120); its contribution is examined in Section 3.9.

Feature importance was also assessed using the native XGBoost metrics. Of the 743 encoded features, 253 were used by the fitted model. Rank correlations with the mean |SHAP| ordering were *ρ* = 0.956 for total gain, 0.928 for weight, 0.737 for gain and −0.540 for cover (all *p* < 10^−19^). *ProximalRiskFactor: Unspecified* ranked first by mean |SHAP|, first by gain, second by total gain and sixth by weight, indicating that its prominence is not specific to the SHAP framework (Supplementary Table S7).

### 3.8 Age-specific contributions to model output

SHAP analysis revealed a non-linear age gradient (Figure 4). Predicted probability of documented prior self-harm peaked in the 35–44 age band (mean SHAP = +0.092), with adolescents (10–17 years, mean SHAP = −0.041) showing a lower mean contribution relative to this peak. The strongest negative mean contribution was observed in older adults (≥65 years, mean SHAP = −0.734, SD = 0.512). Pearson r = −0.442 (*p* < 0.001) and Spearman *ρ* = +0.042 (*p* = 0.002) diverged, reflecting the non-monotonic nature of the relationship. Younger (≤25 years, mean SHAP = −0.065) vs. older (≥55 years, mean SHAP = −0.433) patients showed a statistically significant difference (Mann–Whitney U = 1,203,411, *p* < 10^−300^).

**Figure 4:**
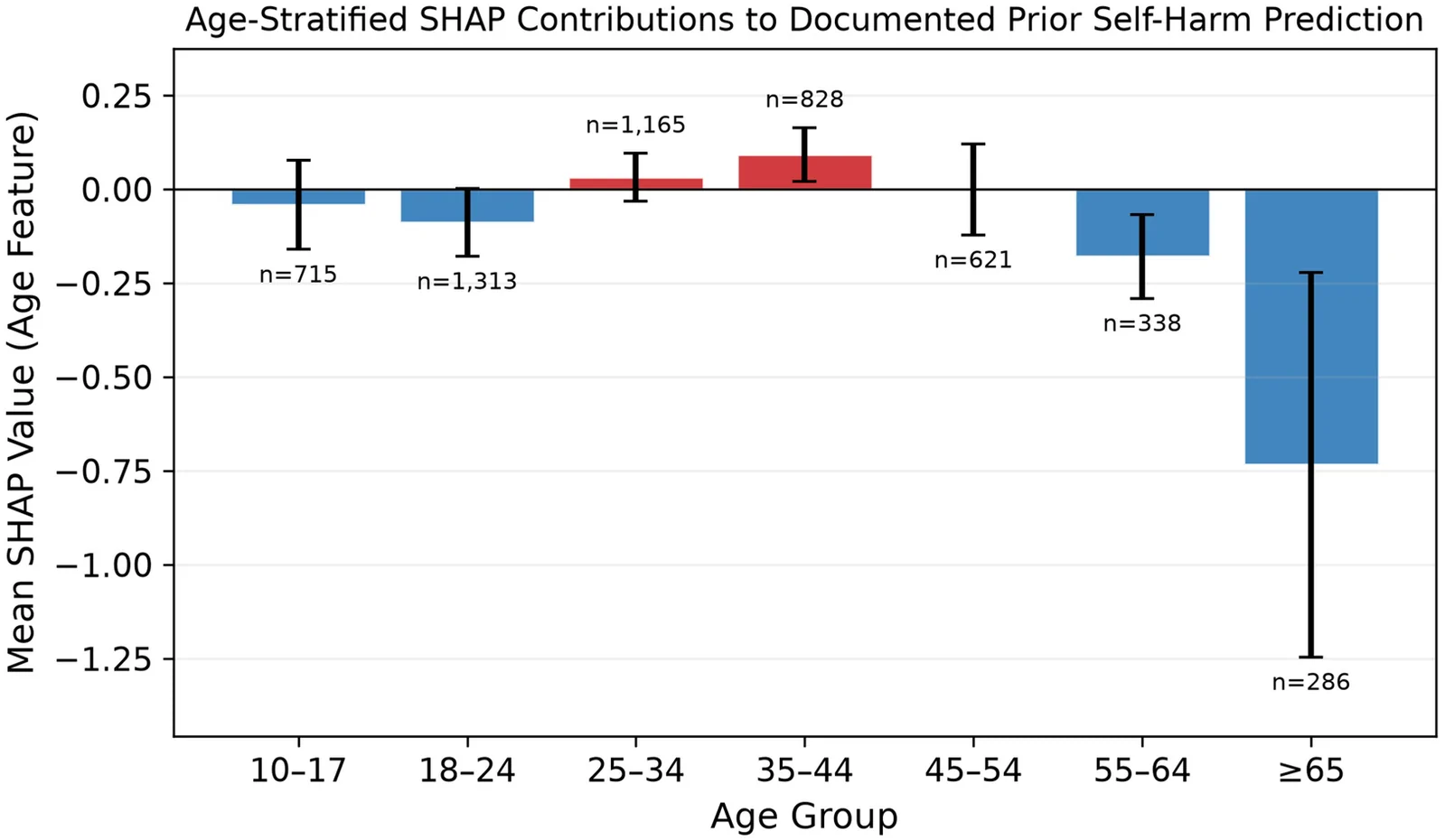
Age-stratified mean SHAP contributions of the AgeOfPatient feature to predicted prior self-harm history. Bars above zero (red) indicate age groups where age increases the predicted probability of documented prior self-harm; bars below zero (blue) indicate age groups where age reduces it. Error bars represent ±1 standard deviation.

### 3.9 COVID-19 period analysis

The binary COVID-19 period indicator, retained as a clinically meaningful temporal predictor after removing Year, showed a large and statistically significant difference in SHAP contributions between pre- and post-2020 presentations (mean SHAP −0.091 vs. +0.215; Mann–Whitney U = 533,190, *p* < 10^−300^; Cliff's *δ* = 0.705) (Supplementary Figure S2). Documented prevalence of prior self-harm history was higher in the post-COVID period (17.5% vs. 13.0%), consistent with published evidence of increased self-harm presentations during and after the pandemic (7).

### 3.10 Fairness analysis

Model performance varied across demographic subgroups (Supplementary Figure S3, and Table 8). By sex, XGBoost performed better in male patients (*n* = 2,545, 320 of which positives; AUC-ROC = 0.818, 95% CI: 0.796–0.839; sensitivity = 84.1%) than in female patients (*n* = 2,765, 405 of which positives; AUC-ROC = 0.783, 95% CI: 0.760–0.804; sensitivity = 78.3%), despite a higher prevalence in females (14.6% vs. 12.6%). However, the confidence intervals were not clearly separated and partially overlapped, precluding strong conclusions regarding sex-related differences in discrimination. By age group, point estimates of discrimination were highest in the 18–24 band (*n* = 1,313, 150 of which positives; AUC-ROC =  0.817, 95% CI: 0.785–0.849) and lowest in patients aged ≥65 years (*n* = 286, 25 of which positives; AUC-ROC = 0.722, 95% CI 0.617–0.822). These intervals overlap substantially, and the difference in discrimination between age bands is therefore not statistically distinguishable. Sensitivity at the operating point shows a clearer pattern: 68.0% in patients aged ≥65 years, against 87.7% in the 35–44 band and 80.8% overall, corresponding to a false negative rate of 32.0% in the oldest group. This performance deficit in older adults is particularly clinically significant given their elevated suicide mortality following self-harm. Adolescents (10–17 years) showed good sensitivity (81.4%) but an elevated false positive rate (39.1%). Confidence interval widths ranged from 0.043 in the largest strata to 0.205 in adults aged ≥65 years and 0.259 in the small stratum of attendances with unrecorded age (*n* = 45), and subgroup comparisons should be read accordingly.

### 3.11 Generalisability assessment

Group-aware 5-fold cross-validation across 14 countries yielded mean AUC-PR = 0.249 ± 0.119 and mean AUC-ROC = 0.658 ± 0.027 (Figure 5). Performance varied across folds: AUC-PR ranged from 0.069 (Fold 1: Sweden) to 0.371 (Fold 5: Cyprus, Czech Republic, Germany, Hungary, Malta, Poland, Romania). The near-chance AUC-PR in the Sweden fold reflects 96.9% Unknown or No classification, which distorts the validation signal. Excluding Sweden yielded substantially improved and more homogeneous AUC-PR (mean 0.294 ± 0.073), with essentially unchanged AUC-ROC (mean 0.652 ± 0.027) (Supplementary Table S9). The gap between test-set performance (AUC-ROC = 0.800) and cross-validation (0.658), a gap of 0.142, reduced from 0.163 in the model with Year, underscores the need for site-specific validation.

**Figure 5:**
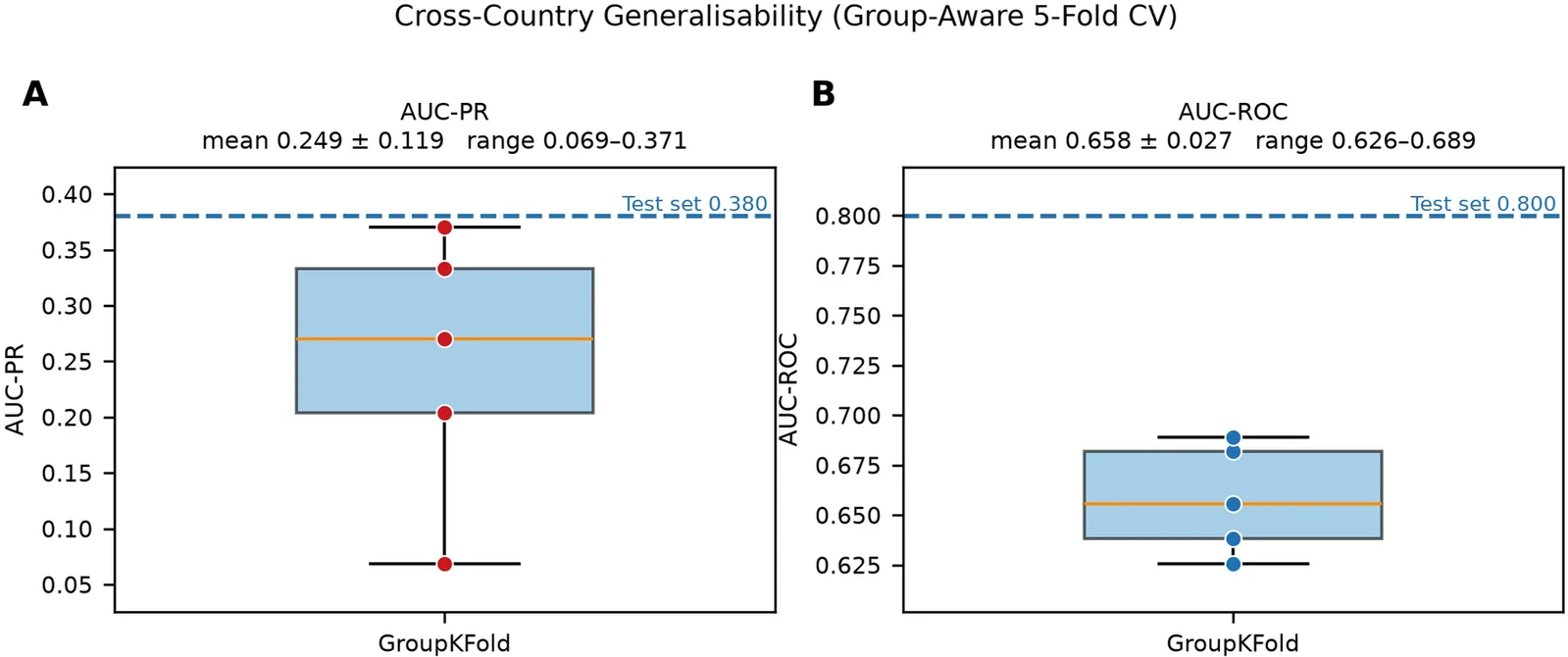
Cross-country generalisability assessed via group-aware 5-fold cross-validation with country as the grouping variable. Box plots show the distribution of **(A)** AUC-PR and **(B)** AUC-ROC across folds, with individual fold values shown as points. Dashed blue lines indicate test-set performance (AUC-PR = 0.380, AUC-ROC = 0.800).

Transfer across time was assessed separately, training on attendances recorded before 2020 (*n* = 22,456) and testing on those from 2020 onwards (*n* = 4,098). This yielded AUC-ROC = 0.674 and AUC-PR = 0.325. Outcome prevalence differed between the two periods (12.6% before 2020 vs. 19.4% after), so the temporal split tests transfer to a population differing in composition as well as in time; the COVID-19 period indicator was necessarily excluded, being constant within the training period. The three designs therefore yield an ordered set of estimates corresponding to progressively demanding deployment scenarios: AUC-ROC = 0.800 within the same system and period, 0.674 across periods within the same countries, and 0.658 across countries.

### 3.12 Sensitivity analysis: exclusion of ProximalRiskFactor

Excluding *ProximalRiskFactor* from the feature set and repeating the full pipeline yielded AUC-ROC = 0.765 and AUC-PR = 0.335 on the independent test set (primary model: 0.800 and 0.380), indicating that discrimination does not depend critically on this potentially circular feature. The corresponding SHAP comparison is discussed below.

### 3.13 Sensitivity analysis: known-outcome-only dataset

Repeating the full modelling pipeline on the Known-outcome-only dataset (*n* = 8,638, prevalence = 42.0%) yielded AUC-ROC = 0.846 and AUC-PR = 0.788, with Brier score = 0.157. SHAP feature ranking showed low stability between the primary and sensitivity models, with only 4 of 10 top features shared. The stable features across both models were *ProximalRiskFactor*: *Psychological/psychiatric condition*, *ObjectInvolved*, *AgeOfPatient*, and *MechanismOfInjury*: *Unknown*—all clinically interpretable signals. The disappearance of *ProximalRiskFactor: Unspecified* from the top 10 in the Known-outcome model confirms that its prominence in the primary model is substantially driven by the Unknown or No classification pattern rather than genuine clinical signal.

## 4 Discussion

### 4.1 What this model actually learns

Before interpreting the model's performance and clinical implications, it is essential to characterise precisely what the model learns and what it does not. The outcome variable (the EU-IDB *PreviousIntentionalSelfHarm* variable) is a documentation event, not a verified clinical fact. It reflects whether prior self-harm history was recorded at the time of ED presentation, which depends on patient disclosure, triage protocols, access to prior records, and jurisdictional coding practices. A model trained to predict this variable therefore learns to recognise presentation patterns associated with documented prior history, it does not learn to detect undisclosed or undocumented history, nor does it predict future episodes.

This distinction has two important implications. First, the model's predictions should be interpreted as a probability that the patient's EU-IDB record is consistent with a documented prior self-harm flag—not as a probability that the patient has clinically significant self-harm history in an absolute sense. Second, some features identified as important by SHAP analysis—most notably *ProximalRiskFactor*: *Unspecified*—likely reflect documentation patterns rather than genuine clinical risk signals. The sensitivity analysis on the Known-outcome-only dataset, in which this feature does not appear among the top predictors, supports this interpretation and should guide clinical readers in weighing individual SHAP contributions.

The clinical rationale for this task remains valid despite these caveats. In routine ED practice, prior self-harm history is one of the strongest predictors of future episodes and completed suicide (1, 2), yet it frequently goes uncommunicated during acute presentations due to patient reluctance, time pressure, or lack of access to prior records (16, 17). A model that flags patients whose injury presentation is strongly consistent with documented prior history—even when that history has not been mentioned—could plausibly prompt targeted clinical inquiry and activate appropriate care pathways. In this sense, the model functions as a pattern-recognition layer that augments, rather than replaces, clinical assessment. It is worth noting that the overlap between clinical signal and documentation pattern is an inherent property of administrative health data and cannot be fully disentangled without access to independent clinical records verified against longitudinal patient histories—a limitation shared by all studies using surveillance-based outcomes.

### 4.2 Documentation artefacts in the feature set

A potential methodological concern arises from the fact that *ProximalRiskFactor*—one of the most important predictors identified by SHAP analysis—is documented during the same ED encounter that generates the *PreviousIntentionalSelfHarm* flag. If the same clinician who records a psychological or psychiatric condition as the proximal risk factor also completes the prior self-harm history variable, these two variables share a common data-generating process, creating a risk of informational circularity. This is distinct from technical data leakage: *ProximalRiskFactor* is a legitimate predictor available at the time of presentation. However, in a real-time deployment scenario where the model is intended to run before or in the absence of a complete clinical assessment, this feature may not yet be populated.

To quantify this dependency, we repeated the full modelling pipeline after excluding *ProximalRiskFactor* from the feature set. The reduced model achieved AUC-ROC = 0.765 and AUC-PR = 0.335, representing decrements of 0.035 and 0.045 respectively compared to the full model. These reductions fall in a moderate range: the model retains clinically useful discrimination without *ProximalRiskFactor*, matching the logistic regression baseline that had access to the full feature set (AUC-ROC = 0.771), but performance is meaningfully lower. SHAP feature ranking showed 8 of 10 top features shared between the full and reduced models: the only features displaced were the two *ProximalRiskFactor* categories themselves, replaced by *TimeOfAttendance* and *SexOfPatient*. The eight shared features—place of occurrence, activity when injured, object involvement pattern, patient age, COVID-19 period, mechanism of injury (two categories), and part of the body injured—are recorded at triage, independently of the clinical assessment that generates the outcome variable.

These findings support two conclusions. First, the model's predictive capacity does not depend critically on *ProximalRiskFactor*: clinical and injury characteristics alone carry substantial discriminative information. Second, any deployment scenario should specify at what point in the encounter the model operates. Excluding *ProximalRiskFactor* alone costs approximately 3.5 percentage points of AUC-ROC; the full time-zero configuration, which also excludes disposition and post-discharge variables, costs 3.8 points (Section 3.5). In both cases the added value of gradient boosting rests on precision rather than on overall discrimination, since the reduced models perform comparably to a linear baseline retaining the full feature set.

Year of attendance was excluded on the same grounds. Including it yielded higher test-set performance (AUC-ROC = 0.810 against 0.800) but lower and less stable cross-validation (0.646, SD 0.030, against 0.658, SD 0.027) the signature of overfitting on country-specific temporal patterns. Year encodes not only epidemiological trends but the progressive change in national data collection protocols evidenced by the decline in missingness from 97.7% in 2008 to 67.9% in 2016. The binary COVID-19 indicator retained in its place captures a clinically meaningful event with a large effect (Cliff's *δ* = 0.705) without encoding that drift, although, being itself a coarse function of calendar time, part of its contribution may still reflect recording completeness rather than epidemiological change.

More broadly, the dominance of “Unspecified proximal risk factor” as the leading predictor (mean |SHAP| = 0.465, and first also by gain) raises the possibility that the model partially learns documentation completeness patterns—distinguishing records where clinicians invested effort in characterising the proximal context from those where the variable was left at its default value—rather than purely clinical signals. This interpretive caveat does not invalidate the model's utility as a screening tool, but it underscores that the model identifies patients whose records look different, which may or may not coincide with patients who are clinically different.

### 4.3 Methodological contribution: the third way for outcome missingness

The most substantive methodological contribution of this study is the formal characterisation and principled resolution of the outcome missingness problem. With 86.2% of records having unknown outcome status, any analytical approach necessarily involves assumptions about the nature of missing data. The naive complete-case approach would yield a highly selected sample with a prevalence of 42.0%, inflated by the documentation mechanism itself: across countries, the sparser the documentation, the higher the proportion of positive cases among documented records (Spearman *ρ* = 0.882, *p* = 0.0003; Section 3.1). The opposite extreme, treating all Unknown records as history-negative across all 18 countries, incorporates 35,913 records from three countries that have no outcome collection mechanism, introducing pure noise that suppresses apparent prevalence to 5.8%.

The Third Way resolves this tension by first identifying the structural component of missingness and excluding countries with 100% Unknown status on quantitative grounds: country membership alone explained 91.1% of missingness variance. Among the remaining 15 countries, the modelling target is defined as a documented positive flag vs. its absence, so that no label is imputed. The resulting dataset is three times larger than the complete-case alternative. The substantive assumption is that documentation is positive-biased—that a positive history, when ascertained, is preferentially recorded—which the country-level relationship reported above supports, and whose impact is bounded by explicit sensitivity scenarios rather than asserted.

We also examined whether the assumption could be relaxed probabilistically, assigning records with an uncoded outcome to positive status with country-specific probabilities. The exercise is not identifiable with these data: because the assignment probability is constant within country, the simulated outcome becomes progressively a function of nationality, and at the extreme scenario recording country alone predicts the simulated label better (AUC-ROC = 0.878) than the full model does (0.865). Given that clinical features recover recording country with 95.3% accuracy, discrimination measured against such labels reflects geographic encoding rather than outcome misclassification (Supplementary Table S10). Treating uncoded records as non-positive is, by contrast, the only rule applied identically across countries, and therefore the only one that does not import geographic structure into the outcome definition.

The sensitivity analysis demonstrated low SHAP feature stability (4/10 top features shared between primary and sensitivity models), with the stable features being those with clearest clinical interpretation. This finding should be reported transparently: the Third Way recoding influences which features appear most important, and researchers using this dataset should verify key findings against the Known-outcome subset before drawing clinical conclusions about specific predictors. Accordingly, clinical interpretation in this study anchors on the features that remained stable across the primary and Known-only models—psychological/psychiatric condition, the object involvement pattern, patient age, and unknown mechanism of injury—rather than on the full importance ranking.

### 4.4 Model performance in context

The primary XGBoost model achieved AUC-ROC = 0.800 (95% CI 0.783–0.816) and AUC-PR = 0.380 (0.351–0.415) on the held-out test set, with NPV = 95.4% and 3.9 model-positive attendances requiring enquiry per documented case identified. It formally outperformed the logistic regression baseline on discrimination and calibration (DeLong *p* = 3.93 × 10^−10^), with the most meaningful improvement on AUC-PR (+0.059); sensitivity at the respective operating points was essentially identical (80.8% vs. 81.0%), and the advantage lay in specificity. A re-specified linear model addressing encoding, scaling, regularisation and non-linearity in age performed essentially identically to the baseline, indicating that the difference is not an artefact of specification. Decision curve analysis confirmed net clinical benefit across clinically relevant threshold probabilities.

These findings are broadly consistent with the growing literature on machine learning for suicide and self-harm risk prediction. Recent systematic reviews have reported pooled AUC-ROC values of 0.80–0.87 for gradient boosting methods predicting self-harm recurrence (20, 28, 31).

### 4.5 Clinical interpretation of precision and number needed to screen

At the optimised threshold of 0.080, the model achieves a positive predictive value of 25.6%, and a number needed to screen of 3.9. The clinical interpretation of these metrics depends critically on the implementation scenario. In the intended use case, a positive model flag would prompt a brief targeted inquiry by the treating clinician, a single additional question about prior self-harm history, requiring approximately 30 s of clinical time. In this context, the “cost” of a false positive is modest: an unnecessary question that carries little procedural burden, though not none—an unwarranted enquiry about a sensitive attribute may affect privacy and the therapeutic relationship, in addition to consuming clinician time. A NNS of 3.9 means that for every 4 patients flagged, one has a documented prior history. We note that this quantity is the reciprocal of the positive predictive value and is not directly comparable with number-needed-to-screen figures reported for universal screening programs, which count attendees screened rather than attendees flagged (14).

If, conversely, a positive flag was to trigger a formal psychiatric consultation or mandatory safety assessment protocol, the cost-benefit calculus would shift substantially: the PPV of 25.6% would mean that approximately three-quarters of triggered consultations would be for patients without documented prior history, potentially straining limited psychiatric liaison resources. The model's value proposition therefore depends on embedding it within a proportionate response framework where the initial action is inquiry rather than intervention. This is consistent with the ED-SAFE trial's finding that screening effectiveness requires coupling with structured but scalable follow-up pathways (10).

The operating point itself is a local configuration decision rather than a property of the model. Optimising for balanced performance (F1) shifts the threshold to 0.250, reducing false positives from 1,707 to 899 at the cost of 134 documented cases; a precision-weighted objective (F0.5) shifts it to 0.300, with 379 false positives but fewer than 40% of documented positives identified. Settings where alert burden is the binding constraint may reasonably prefer the former. Settings where alert burden is the binding constraint may reasonably prefer the former; the appropriate choice depends on staffing, alerting infrastructure and the clinical pathway triggered by a positive output.

### 4.6 Transportability across countries and over time

The gap between test-set performance (AUC-ROC = 0.800) and group-aware cross-validation (mean AUC-ROC = 0.658), a difference of 0.142, warrants explicit characterisation. Although country identifiers were excluded from the feature set to prevent direct geographical data leakage, the clinical features themselves encode substantial geographic information through country-specific coding practices. To quantify this, we trained a multiclass classifier to predict the recording country from the same feature set used in the primary model. The classifier achieved 95.3% accuracy across 15 countries, a 14.3-fold improvement over chance level (6.7%). The features contributing most to country identification were specific object codes (*MainObject*), activity categories, and mechanism of injury codes, reflecting national differences in IDB coding practices rather than genuine clinical variation.

This finding has two implications. First, it provides a plausible mechanistic explanation for the test-set vs. cross-validation gap: when the test set shares countries with the training set, the model benefits from country-specific coding patterns unavailable in the leave-country-out design. Second, it underscores that the test-set AUC-ROC of 0.800 is best interpreted as within-system performance—the discrimination achievable when the model is deployed within the same national coding context in which it was trained.

A third estimate situates these two. Training on attendances before 2020 and testing on those from 2020 onwards yielded AUC-ROC = 0.674, between the within-system and cross-country values. The three designs therefore correspond to progressively more demanding deployment scenarios: 0.800 within the same system and period, 0.674 across periods within the same countries, and 0.658 across countries. All three are clinically relevant, but they answer different questions, and the appropriate expectation for a given implementation depends on which kind of transfer it involves. This pattern is consistent with evidence from other multinational settings: models transferred between healthcare systems without local adaptation have shown discrimination falling to near-chance levels, recoverable only through site-specific fine-tuning, with the loss attributed to differences in annotation practice and data collection tools rather than to clinical divergence (32).

Calibration showed a comparable pattern. On the test set as a whole the model was well calibrated (calibration intercept +0.022, slope 0.931, against reference values of 0 and 1), but stratified analysis revealed heterogeneity across national contexts (Supplementary Figure S4). Calibration quality tracked test-set size: the four countries contributing more than 400 test-set records—Sweden (MACE = 0.019, *n* = 1,792), Turkey (0.025, *n* = 1,131), Latvia (0.041, *n* = 989) and Luxembourg (0.035, *n* = 441)—all showed mean absolute calibration error below 0.05. An intermediate group fell between 0.05 and 0.10: Italy (0.066, *n* = 130), Austria (0.072, *n* = 85), Germany (0.073, *n* = 154) and Malta (0.086, *n* = 404). Only the Czech Republic (0.135, *n* = 88) and Spain (0.161, *n* = 57) exceeded 0.10, both on small samples that limit the reliability of these estimates. We note that these cut-points are pragmatic descriptive thresholds adopted for interpretation rather than validated standards, and no conclusion here depends on them.

The miscalibration was directional rather than random. Calibration intercepts ordered almost exactly with national outcome prevalence: the model under-predicted risk in high-prevalence countries (Spain +0.811, Czech Republic +0.495, Italy +0.277, Malta +0.225) and slightly over-predicted at the low-prevalence extreme (Sweden −0.177, Luxembourg −0.162). This is the expected behaviour of a calibration function fitted on pooled data with an aggregate prevalence of 13.7%: predictions are compressed towards that central value, and the compression is most visible where national prevalence departs furthest from it. The slopes add a distinction the MACE does not convey—Austria (2.11) and Germany (1.57) show predicted probabilities that are too tightly clustered, whereas Italy (0.51) and the Czech Republic (0.56) show the opposite—indicating that different national contexts require different corrections rather than a single adjustment.

Any deployment in a specific national healthcare system should therefore include a local recalibration step using prospectively collected data from that system. As a practical consideration, calibration estimates from very small country-specific samples are unstable, and on the order of 200–300 observations including at least 50 positive outcomes would be needed for reasonable stability; we offer this as guidance rather than as a methodological requirement. The isotonic framework used here is directly adaptable to this purpose, requiring only a validation dataset from the deployment site—although recalibration addresses the scaling of predicted probabilities within the population on which it is fitted and does not remove structural differences in outcome prevalence, coding practice or documentation culture between healthcare systems.

Finally, these findings suggest that harmonisation of IDB coding practices across countries—particularly for mechanism of injury, activity and object codes—could reduce implicit geographic encoding and improve cross-national transportability. Whether such harmonisation is feasible within the current EU-IDB governance framework, and whether it would preserve clinically meaningful variation, are open questions for future collaborative work.

### 4.7 Subgroup variation and equity considerations

Model output varied non-linearly with age, peaking in the 35–44 band rather than in adolescents, a pattern that differs from some prior work and may reflect the compositional differences of the Third Way dataset relative to complete-case approaches. Older adults (≥65 years) showed the strongest negative mean signed SHAP contribution (−0.734). This is consistent with literature reporting lower repetition of self-harm at older ages (1, 33), although in this dataset it may equally reflect later age of onset and shorter documented histories—that is, a lower probability of having an episode recorded rather than a lower underlying risk. As throughout, these values describe the behaviour of the fitted model under a specific outcome definition and should not be read as evidence about the epidemiology of self-harm repetition.

By sex, discrimination was lower in female than in male patients (AUC-ROC 0.783, 95% CI 0.760–0.804, vs. 0.818, 0.796–0.839), a difference supported by non-overlapping confidence intervals and notable given the higher prevalence among women in this dataset (14.6% vs. 12.6%). The mechanisms may reflect differences in injury morphology, documentation patterns or disclosure behaviour not fully captured by the available features (16).

Differences in discrimination across age bands are less certain. The point estimate for adults aged ≥65 years (AUC-ROC 0.722) is the lowest observed, but with 286 attendances and 25 positive cases its confidence interval (0.617–0.822) overlaps substantially with that of the best-performing band (18–24: 0.817, 0.785–0.849), and the difference is not statistically distinguishable. The equity concern in this group rests instead on sensitivity at the operating point, which is more stable and more directly consequential: 68.0% against 87.7% in the 35–44 band and 80.8% overall, a false negative rate of 32.0%. Older adults face elevated suicide mortality following self-harm (5), and a one-in-three miss rate is difficult to accept in a tool intended to support clinical safety. Threshold adjustment and subgroup-specific recalibration are the feasible near-term mitigations; the size of this stratum would not currently support training a dedicated geriatric model.

These disparities need not reflect model behaviour alone. Because the outcome is itself subject to differential documentation, and because the analytical dataset rests on an explicit outcome-construction assumption, observed differences across strata may arise from genuine differences in performance, from differences in recording practice, from differential outcome missingness, or from variation in healthcare utilisation and assessment patterns. The last of these deserves particular attention. If documentation completeness partly tracks prior contact with the health system, the model will assign systematically lower predictions to patients whose index attendance is their first substantive contact—precisely the group for whom a prior episode is least likely to be on record and, arguably, most likely to be missed clinically. The EU-IDB contains no measure of prior healthcare contact, so we cannot adjust for or quantify this effect.

Algorithmic fairness assessment should be a pre-condition for any real-world deployment, and stratified performance monitoring maintained prospectively (18). We note that the present assessment constitutes subgroup performance stratification—a necessary but not sufficient component of a comprehensive fairness audit, which would additionally require formal fairness criteria (for example equalised odds or calibration within groups) and richer sociodemographic variables than the EU-IDB provides.

### 4.8 The critical role of post-identification intervention

It is essential to emphasise that identification of prior self-harm history is a necessary but not sufficient condition for improved patient outcomes. The ED-SAFE trial demonstrated that universal screening successfully increased detection rates but did not reduce subsequent suicidal behaviour unless coupled with structured post-screening interventions including safety planning and systematic follow-up telephone contacts, which produced a 5% absolute (20% relative) reduction in the proportion of patients attempting suicide and 30% fewer total suicide attempts (incidence rate ratio 0.72, 95% CI 0.52–1.00) (10). A positive ML-based flag must therefore trigger appropriate clinical responses to translate detection into benefit. This model should be understood as a decision support aid that prompts enquiry about documented prior history, not as a replacement for clinical assessment or structured intervention.

### 4.9 Ethical considerations for implementation

Although this study is a retrospective methodological evaluation and the model is not proposed for immediate use, several considerations would condition any implementation.

The output is a decision-support signal, not a determination. A positive prediction should prompt a targeted enquiry about prior episodes in a patient already being treated for self-harm—a question that is in any case clinically appropriate in this population—and never a treatment decision, a diagnostic classification or an automated risk stratification. Clinician judgement always takes precedence, and clinicians must be able to disregard the output without justification; responsibility for assessment remains with the treatment team.

A negative output is uninformative rather than reassuring. At the selected operating point 19.2% of documented positive cases are not flagged, and among adults aged ≥65 years the proportion is 32.0%. Any implementation that allowed a negative prediction to justify omitting clinical enquiry would invert the purpose of the tool.

False positives are not costless. An unwarranted enquiry about self-harm history touches on a sensitive attribute and may affect privacy, dignity and therapeutic relationship, in addition to consuming clinician time. This bears directly on the choice of operating threshold discussed in Section 4.5.

The subgroup disparities documented here carry an equity implication. Uniform deployment would distribute the benefit of the tool unevenly across age groups, in a direction opposite to suicide mortality risk following self-harm, and—to the extent that documentation completeness tracks prior healthcare contact—would disadvantage patients whose index attendance is their first substantive contact with the health system. Prospective monitoring stratified by subgroup should be a condition of implementation rather than a subsequent audit.

Finally, because the outcome is a documented flag rather than a verified clinical history, the model must not be used in any clinical, administrative or forensic context in which its output could be read as establishing that a patient did or did not previously self-harm.

### 4.10 Study limitations

Several limitations merit consideration. First, the outcome variable captures documented history at ED presentation, which may be incomplete or inconsistently recorded: the model identifies patterns associated with documented prior history but cannot distinguish true first-time presentations from cases where history was present but not disclosed or recorded. Importantly, misclassification in Known records (patients recorded as “No” who in fact have undisclosed prior history) represents non-differential misclassification that attenuates discrimination estimates toward the null. Observed model performance may therefore be conservative relative to what would be achieved with perfectly ascertained outcomes. Even among Known records, misclassification of prior self-harm history cannot be excluded due to reliance on patient disclosure and clinical documentation at a single time point. Second, while the country-driven structure of missingness is supported empirically, we cannot exclude residual within-country MNAR components driven by unobserved factors such as patient disclosure propensity, facility-level triage variation, or differences in access to prior medical records within a country. This residual MNAR component is not quantifiable with the available data. More broadly, the missingness assumptions—structural, country-driven missingness across countries, and documentation-positive MNAR within countries—are supported by, but cannot be verified from, the observed data. Third, the Unknown or No classification introduces systematic misclassification that affects feature importance rankings, as demonstrated by the low SHAP stability in the Known-outcome-only sensitivity analysis (4/10 top features shared). Fourth, the operating threshold of 0.080 was optimised on the aggregated multinational dataset and may not be stable across deployment contexts with substantially different outcome prevalences. The F2 objective is moreover flat in the neighborhood of its optimum: isotonic calibration produces a step function, and thresholds between 0.080 and 0.100 yield near-identical classifications on the test set (Table 1). The selected value should therefore be read as one point within a region of equivalent operating characteristics, not as a precise cut-off, and country-specific threshold recalibration is recommended before operational use. Fifth, the gap between test-set (AUC-ROC = 0.800) and cross-validation performance (mean = 0.658)—a difference of 0.142—underscores the need for site-specific validation before deployment. Relatedly, no external validation on fully independent data was performed; leave-country-out cross-validation approximates, but does not replace external validation. Relatedly, the EU-IDB record structure contains no patient identifier: records represent attendances rather than individuals, and repeated attendances by the same person cannot be linked, so we could not ensure that all attendances of a given patient fell within the same partition. Because prior self-harm is by definition more common among repeat attenders, any such overlap would tend to inflate rather than deflate apparent performance. Sixth, fairness assessment was limited to variables available in the EU-IDB dataset; socioeconomic status, migration background, and healthcare access—which may be relevant sources of algorithmic bias—were not evaluable and represent important targets for future fairness audits using enriched datasets. The logistic regression baseline received the identical, tree-friendly input representation as XGBoost—unstandardised numerical features and an integer-coded *ObjectInvolved* rather than one-hot encoding—so the primary comparison should be read as XGBoost vs. a linear model under identical preprocessing. We tested whether this disadvantaged the baseline by re-specifying it with one-hot encoding of *ObjectInvolved*, standardised predictors, a cubic spline basis for age and cross-validated regularisation: performance was essentially unchanged (AUC-ROC 0.7714 vs. 0.7710), and the difference from XGBoost remained significant (DeLong Z = 6.34, *p* = 2.3 × 10^−10^). Eighth, the retrospective design limits our ability to assess real-world implementation feasibility or clinical impact on patient outcomes. Ninth, the reliance on EU-IDB coding schemes means that feature importance findings may have limited face validity without access to detailed code definitions; researchers are referred to the EU-IDB coding manual for interpretation of specific mechanism and place codes.

Future work should include: external validation on data from healthcare systems not represented in the EU-IDB network, and prospective validation studies assessing real-world clinical workflow impact and patient outcomes; investigation of subgroup-specific threshold recalibration and model adaptation for older adults, where current sensitivity is clinically inadequate; fairness audits incorporating socioeconomic, migration, and healthcare access dimensions using enriched datasets; exploration of whether COVID-19 period encoding remains stable as new data collection waves extend beyond 2023; head-to-head comparison with simpler rule-based clinical screening tools currently in use; and examination of whether this approach can be extended to predict future episodes when longitudinal follow-up data become available.

## 5 Conclusion

This study demonstrates that injury presentation patterns routinely recorded in ED surveillance systems carry substantial information about documented prior self-harm history among patients presenting with intentional self-harm, and that an interpretable machine learning model trained on these patterns can achieve clinically meaningful discrimination across 15 European countries. The Third Way classification strategy—grounded in formal quantitative characterisation of the missingness mechanism and applied transparently—provides a transparent framework for settings with comparably structured outcome unknown information for future research using EU-IDB data. The deliberate exclusion of Year of attendance from the feature set, supported by evidence that this variable encodes documentation artefacts more than clinical signal, yielded a model with better cross-national transportability at a modest cost in test-set performance.

Four boundaries of this approach must be clearly understood by clinicians, researchers, and policymakers considering implementation. First, the model predicts the probability that a patient's EU-IDB record carries a documented prior self-harm flag—not the absolute probability of clinical self-harm history. Its outputs should trigger clinical inquiry, not substitute for it. Moreover, the performance reported for the full-encounter model reflects information accumulated across the whole episode; a real-time implementation operating at triage would achieve the lower performance of the time-zero configuration (AUC-ROC 0.762). Second, cross-country performance variability (AUC-PR 0.069–0.371 across validation folds) means that no single threshold or calibration can be applied uniformly: local validation and recalibration are prerequisites for responsible deployment in any individual healthcare system. Third, the performance deficit in older adults (sensitivity 68.0%, FNR 32.0%) in a population at elevated suicide risk is a critical gap that must be explicitly addressed before implementation. Fourth, evidence from large-scale ED intervention trials demonstrates that risk identification alone does not improve outcomes: integration within structured care pathways including safety planning, treatment engagement, and systematic follow-up is essential to translate model outputs into patient benefit (10).

With appropriate governance, continuous monitoring, fairness auditing, and embedding within structured post-identification care pathways, interpretable machine learning applied to routine injury surveillance data represents a promising decision support layer for enhancing early identification of patients with prior self-harm history in emergency settings across diverse European healthcare contexts.

## Data Availability

The data analysed in this study are subject to the following licenses/restrictions: the European Injury Database (EU-IDB) is a restricted-access surveillance database. Data are contributed by national injury surveillance systems under data-sharing agreements and cannot be redistributed by the authors. Aggregated data supporting the findings of this study are provided in the Supplementary Material. Requests to access the datasets should be directed to the corresponding author.
