# Supplementary material for: Predicting documented prior intentional self-harm in emergency departments: an interpretable gradient boosting approach using European injury surveillance data
Source: Front Digit Health. 2026 Oct 14;8:1964834. doi: 10.3389/fdgth.2026.1964834 (PMC13713467; doi:10.3389/fdgth.2026.1964834)
Supplement: Supplementary file 1 [file Datasheet1.docx]

Supplementary Material

# 1 Supplementary Figures and Tables

## 1.1 Supplementary Figures

**Supplementary Figure 1.** Decision Curve Analysis comparing XGBoost (solid blue) and Logistic Regression (dashed green) against default strategies of treating all patients (dotted grey) and treating none (solid black). The x-axis represents the threshold probability at which a clinician would consider a positive screen actionable; the y-axis represents net clinical benefit. Both models provide positive net benefit over default strategies across a clinically relevant range of threshold probabilities (approximately 0.01–0.47). XGBoost consistently outperforms Logistic Regression, with the advantage most pronounced at threshold probabilities between 0.10 and 0.30 — the range most likely to be adopted in ED screening workflows where high sensitivity is prioritised over precision. The y-axis is truncated at −0.05 to make the informative region legible; the Treat All curve continues to decline beyond this range.

**Supplementary Figure 2.** Distribution of SHAP contributions for the COVID_period binary feature, comparing pre-COVID (2008–2019) and post-COVID (2020–2023) presentations on the independent test set. Violin plots show the density of individual SHAP values; horizontal lines indicate median and interquartile range. Pre-COVID presentations receive predominantly negative SHAP contributions (mean=−0.091), shifting predictions away from documented prior self-harm history, while post-COVID presentations receive substantially higher and more variable positive contributions (mean=+0.215). The difference is large (Cliff's δ=0.705, Mann-Whitney U=549,493, p<10⁻³⁰⁰), consistent with higher documented prevalence in the post-COVID period (17.5% vs 13.0%) and reflecting changes in ED presentation patterns during and after the pandemic.

**Supplementary Figure 3.** Equalized odds assessment across demographic subgroups on the independent test set. Bars show sensitivity (true positive rate, blue) and false positive rate (red) stratified by sex (left panel) and age group (right panel). By sex, sensitivity is comparable (Female 78.3%, Male 84.1%), with slightly higher FPR in females. By age group, sensitivity is highest in adolescents (10–17: 81.4%) and in the 35–44 band (87.7%), and drops markedly in older adults (≥65 years: 68.0%), identifying this subgroup as a critical equity concern for deployment. Older adults also show the lowest false positive rate (28.4% against a range of 28.4–43.3% across age groups): the co-occurrence of low sensitivity and low FPR indicates that predicted probabilities are systematically shifted downwards in this group, so that part of the deficit may be recoverable through age-specific threshold adjustment. Discrimination in this subgroup is also the lowest observed (AUC-ROC 0.722), although the confidence interval is wide and the difference from other age bands is not statistically distinguishable. The 'Unknown' age stratum comprises 45 records with unrecorded age and should not be interpreted.

**Supplementary Figure 4.** Country-stratified calibration curves for the XGBoost model on the independent test set. Each panel shows the relationship between mean predicted probability (x-axis) and observed fraction of history-positive cases (y-axis) for a single country, with the dashed diagonal representing perfect calibration. Country sample size and local prevalence are indicated in the panel titles. Latvia (n=989, MACE=0.041), Turkey (n=1,131, MACE=0.025), and Sweden (n=1,792, MACE=0.019) demonstrate well-calibrated predictions. Spain (n=57, MACE=0.161) and Czech Republic (n=88, MACE=0.135) show substantial miscalibration attributable to small sample sizes. Countries with fewer than 50 test-set observations (Cyprus, Greece, Hungary, Poland, Romania) were excluded from this analysis.

## 1.2 Supplementary Tables

**Table 1.** Sensitivity analysis under missing-not-at-random (MNAR) assumptions. Implied overall prevalence of history-positive cases if a given percentage of the 53,829 Unknown records were true positives. Implied prevalences are computed over the full 18-country dataset (N=62,467); the prevalence of the Third Way analytical dataset (13.7%, computed over N=26,554) is reported in the last row for reference only and is not directly comparable with the implied whole-dataset prevalences.

| **MNAR Scenario** | **Unknown true positives (%)** | **Implied N (Yes)** | **Implied prevalence (%)** |
| --- | --- | --- | --- |
| Observed (Known only) | — | 3,625 | 5.8 |
| 5% MNAR | 5 | 6,316 | 10.1 |
| 10% MNAR | 10 | 9,007 | 14.4 |
| 20% MNAR | 20 | 14,390 | 23.0 |
| 30% MNAR | 30 | 19,773 | 31.7 |
| 50% MNAR | 50 | 30,539 | 48.9 |
| Third Way analytical dataset | — | 3,625 | 13.7 |

**Table 2.** SHAP contribution of year of attendance, by calendar year, in a preliminary model retaining Year in the feature set. Values are computed on the independent test set (n = 5,311). Documented prevalence is the proportion of attendances with a positive documented flag in that year. The mean contribution is negative in every year from 2008 to 2015 and positive in every year from 2016 onwards, a single sign reversal across the sixteen-year period.

| **Year** | **N** | **Mean SHAP** | **Median SHAP** | **Mean \|SHAP\|** | **Documented prevalence** |
| --- | --- | --- | --- | --- | --- |
| 2008 | 200 | −0.509 | −0.531 | 0.512 | 5.0% |
| 2009 | 206 | −0.448 | −0.447 | 0.450 | 9.2% |
| 2010 | 208 | −0.064 | −0.075 | 0.241 | 13.5% |
| 2011 | 273 | −0.140 | −0.123 | 0.225 | 11.4% |
| 2012 | 395 | −0.154 | −0.127 | 0.180 | 14.2% |
| 2013 | 865 | −0.475 | −0.444 | 0.475 | 13.6% |
| 2014 | 524 | −0.604 | −0.564 | 0.604 | 9.4% |
| 2015 | 718 | −0.581 | −0.632 | 0.581 | 10.0% |
| 2016 | 403 | +0.354 | +0.364 | 0.355 | 22.8% |
| 2017 | 268 | +0.430 | +0.455 | 0.430 | 21.6% |
| 2018 | 247 | +0.052 | +0.029 | 0.080 | 12.2% |
| 2019 | 202 | +0.041 | +0.015 | 0.077 | 10.9% |
| 2020 | 167 | +0.039 | +0.009 | 0.100 | 5.4% |
| 2021 | 212 | +0.070 | +0.066 | 0.186 | 20.3% |
| 2022 | 238 | +0.272 | +0.352 | 0.471 | 17.2% |
| 2023 | 185 | +0.247 | +0.289 | 0.326 | 25.4% |

*Period means: −0.428 for 2008–2015 and +0.214 for 2016–2023. The transition coincides with the year in which outcome missingness reached its minimum across the study period (67.9%; see Section 3.1) and documented prevalence rose to 22.8% before returning to 12.2% by 2018.*

**Table 3.** Country weights applied during model training. Weights are the inverse of each country's training-set frequency, normalised so that the mean of the inverse weights is 1, and clipped to [0.5, 5.0]. Countries are ordered by training-set contribution.

| **Country** | **Training records** | **Raw weight (1/n)** | **Normalised** | **Applied (clipped)** |
| --- | --- | --- | --- | --- |
| Sweden | 5,446 | 0.00018 | 0.003 | 0.500 |
| Turkey | 3,121 | 0.00032 | 0.005 | 0.500 |
| Latvia | 2,990 | 0.00033 | 0.005 | 0.500 |
| Luxembourg | 1,383 | 0.00072 | 0.012 | 0.500 |
| Malta | 1,343 | 0.00074 | 0.012 | 0.500 |
| Germany | 466 | 0.00215 | 0.035 | 0.500 |
| Italy | 355 | 0.00282 | 0.045 | 0.500 |
| Austria | 252 | 0.00397 | 0.064 | 0.500 |
| Czech Republic | 248 | 0.00403 | 0.065 | 0.500 |
| Spain | 202 | 0.00495 | 0.080 | 0.500 |
| Romania | 85 | 0.01176 | 0.189 | 0.500 |
| Hungary | 30 | 0.03333 | 0.537 | 0.537 |
| Cyprus | 5 | 0.20000 | 3.219 | 3.219 |
| Poland | 3 | 0.33333 | 5.365 | 5.000 |
| Greece | 3 | 0.33333 | 5.365 | 5.000 |

*Clipping is binding for thirteen of the fifteen countries. Eleven fall at the lower bound of 0.5, including every country contributing more than eighty training records, so that Sweden (5,446 records) and Romania (85) carry identical weight. Two fall at the upper bound. The applied weights therefore do not rebalance large against small contributors; their net effect is a fivefold amplification of Poland and Greece, which together contribute six training records.*

**Table 4.** Outcome missingness by recording country. Countries are sorted by descending percentage of Unknown outcome status. Countries with 100% Unknown (Netherlands, Denmark, Slovenia) were excluded from the analytical dataset. N (Yes) = documented prior self-harm history; N (No) = no documented prior history; N (Unknown) = outcome status not recorded.

| **Country** | **N** | **N (Yes)** | **N (No)** | **N (Unknown)** | **% Unknown** | **% Yes** | **N (Known)** | **% Yes among Known** |
| --- | --- | --- | --- | --- | --- | --- | --- | --- |
| Netherlands | 28,827 | 0 | 0 | 28,827 | 100.0 | 0.0 | 0 | — |
| Denmark | 4,799 | 0 | 0 | 4,799 | 100.0 | 0.0 | 0 | — |
| Slovenia | 2,287 | 0 | 0 | 2,287 | 100.0 | 0.0 | 0 | — |
| Sweden | 8,950 | 270 | 8 | 8,672 | 96.9 | 3.0 | 278 | 97.1 |
| Latvia | 5,009 | 953 | 244 | 3,812 | 76.1 | 19.0 | 1,197 | 79.6 |
| Luxembourg | 2,291 | 411 | 155 | 1,725 | 75.3 | 17.9 | 566 | 72.6 |
| Germany | 767 | 193 | 32 | 542 | 70.7 | 25.2 | 225 | 85.8 |
| Hungary | 42 | 12 | 4 | 26 | 61.9 | 28.6 | 16 | 75.0 |
| Austria | 424 | 69 | 99 | 256 | 60.4 | 16.3 | 168 | 41.1 |
| Italy | 610 | 200 | 97 | 313 | 51.3 | 32.8 | 297 | 67.3 |
| Czech Republic | 407 | 98 | 141 | 168 | 41.3 | 24.1 | 239 | 41.0 |
| Spain | 330 | 137 | 66 | 127 | 38.5 | 41.5 | 203 | 67.5 |
| Turkey | 5,358 | 640 | 3,012 | 1,706 | 31.8 | 11.9 | 3,652 | 17.5 |
| Greece | 7 | 0 | 5 | 2 | 28.6 | 0.0 | 5 | 0.0 |
| Cyprus | 8 | 4 | 2 | 2 | 25.0 | 50.0 | 6 | 66.7 |
| Malta | 2,199 | 594 | 1,055 | 550 | 25.0 | 27.0 | 1,649 | 36.0 |
| Poland | 4 | 1 | 2 | 1 | 25.0 | 25.0 | 3 | 33.3 |
| Romania | 148 | 43 | 91 | 14 | 9.5 | 29.1 | 134 | 32.1 |
| Total | 62,467 | 3,625 | 5,013 | 53,829 | 86.2 | 5.8 | 8,638 | 42.0 |

*N (Known) is the number of records with a coded outcome (Yes or No); % Yes among Known is the proportion of positive cases among them, and is undefined for the three countries with no coded outcome. Values based on fewer than 100 coded records (Hungary, Greece, Cyprus, Poland) are unstable and were excluded from the correlation reported in Section 3.1.*

**Table 5.** Logistic regression models predicting Unknown vs Known outcome status. Model A uses country membership alone; Model B uses clinical and demographic variables without country; Model C combines both. McFadden's pseudo-R² and AUC-ROC are reported. Country membership alone explains 91.1% of the full-model pseudo-R² (0.537/0.589), indicating a predominantly country-driven missingness structure.

| **Model** | **Predictors** | **Pseudo-R²** | **AUC** |
| --- | --- | --- | --- |
| Model A | Country only | 0.537 | 0.948 |
| Model B | Clinical variables only  *(Year, Sex, Age group, ProximalRiskFactor)* | 0.341 | 0.892 |
| Model C | Full (Country + Clinical) | 0.589 | 0.963 |

*Country explains 91.1% of full-model pseudo-R² (Model A pseudo-R² / Model C pseudo-R² = 0.537/0.589).*

**Table 6.** Top 20 features ranked by mean absolute SHAP value on the independent test set. SHAP values were computed on the full independent test set (n=5,311) using TreeExplainer. Raw IDB-FDS variable codes are shown in parentheses where applicable. Features are one-hot encoded dummy variables except for *AgeOfPatient*, *ObjectInvolved*, and COVID_period. The full model uses 743 encoded features derived from 14 original predictors.

| **Rank** | **Feature** | **Mean \|SHAP\|** |
| --- | --- | --- |
| 1 | *ProximalRiskFactor*: Unspecified | 0.465 |
| 2 | *ProximalRiskFactor*: Psychological/psychiatric condition | 0.230 |
| 3 | *PlaceOfOccurrence*: Private home | 0.208 |
| 4 | *ActivityWhenInjured*: Leisure/unspecified | 0.194 |
| 5 | *ObjectInvolved* (pattern of populated object/substance layers, 12 categories) | 0.140 |
| 6 | *AgeOfPatient* | 0.128 |
| 7 | *COVID_period* | 0.120 |
| 8 | *MechanismOfInjury*: Unknown | 0.087 |
| 9 | *PartOfTheBodyInjured1*: Multiple/systemic | 0.075 |
| 10 | *MechanismOfInjury*: Cutting/piercing sharp object | 0.068 |
| 11 | *TimeOfAttendance*: Unknown | 0.066 |
| 12 | *ProximalRiskFactor:* Conflict in relationship | 0.052 |
| 13 | *SexOfPatient*: Female | 0.052 |
| 14 | *ActivityWhenInjured*: Unknown | 0.052 |
| 15 | *TypeOfInjury1*: Open wound | 0.051 |
| 16 | *ActivityWhenInjured*: Education | 0.050 |
| 17 | *MechanismOfInjury*: Fall — same level | 0.050 |
| 18 | *ActivityWhenInjured*: Other specified | 0.048 |
| 19 | *MechanismOfInjury*: Cutting/piercing other | 0.048 |
| 20 | *TypeOfInjury1*: Fracture | 0.045 |

**Table 7**. Comparison of SHAP-based and native XGBoost feature importance metrics. The twenty features with the highest mean |SHAP| are shown, with their values and ranks under gain (average improvement per split), total gain (cumulative improvement) and weight (number of splits). Ranks are computed among the 253 features actually used by the fitted model. Feature names are abbreviated as in Figure 2.

| **Feature** | **mean \|SHAP\|** | **Gain** | **Rank** | **Total gain** | **Rank** | **Weight** | **Rank** |
| --- | --- | --- | --- | --- | --- | --- | --- |
| *Risk factor*: unspecified | 0.465 | 27.41 | 1 | 4,741 | 2 | 173 | 6 |
| *Risk factor*: psych. condition | 0.230 | 16.51 | 4 | 2,789 | 4 | 169 | 7 |
| *Place*: Living room | 0.208 | 14.05 | 6 | 2,107 | 6 | 150 | 9 |
| *Activity*: Unspecified leisure or play | 0.194 | 21.39 | 3 | 1,240 | 9 | 58 | 27 |
| Object involvement pattern | 0.140 | 5.94 | 78 | 3,186 | 3 | 536 | 2 |
| Age of patient | 0.128 | 4.45 | 147 | 5,398 | 1 | 1,214 | 1 |
| COVID-19 period | 0.120 | 13.09 | 8 | 2,108 | 5 | 161 | 8 |
| *Mechanism*: Unknown | 0.087 | 11.87 | 12 | 926 | 11 | 78 | 21 |
| *Body part*: Whole body affected | 0.075 | 8.24 | 25 | 783 | 12 | 95 | 15 |
| *Mechanism*: Poisoning by solid substance | 0.068 | 7.51 | 34 | 736 | 14 | 98 | 12 |
| *Time*: Unknown | 0.066 | 21.52 | 2 | 1,421 | 8 | 66 | 23 |
| *Risk factor*: relationship conflict | 0.052 | 7.21 | 40 | 707 | 15 | 98 | 12 |
| *Sex*: Female | 0.052 | 3.91 | 184 | 946 | 10 | 242 | 4 |
| *Activity*: Unknown | 0.052 | 5.47 | 92 | 492 | 24 | 90 | 19 |
| *Injury type*: Open wound | 0.051 | 6.62 | 54 | 629 | 17 | 95 | 15 |
| *Activity*: Other specified unpaid work | 0.050 | 10.55 | 16 | 485 | 25 | 46 | 33 |
| *Mechanism*: Contact with static object | 0.050 | 9.96 | 19 | 418 | 29 | 42 | 37 |
| *Activity*: Other specified activity | 0.048 | 4.41 | 154 | 775 | 13 | 176 | 5 |
| *Mechanism*: Poisoning by liquid substance | 0.048 | 10.66 | 15 | 522 | 22 | 49 | 31 |
| *Injury type*: Fracture | 0.045 | 12.75 | 9 | 510 | 23 | 40 | 40 |

*Rank correlations with the mean |SHAP| ordering, computed over all 253 used features, were ρ = 0.956 for total gain, 0.928 for weight, 0.737 for gain and −0.540 for cover (all p < 10^-19^). Concordance is strongest for aggregate measures. Divergences are informative rather than contradictory: continuous features available at every split (age of patient, object involvement pattern) rank first and second by weight and total gain but lower by gain, since they accumulate many splits of individually modest improvement, whereas features such as time of attendance coded as unknown rank second by gain but twenty-third by weight, being used rarely but decisively*

**Table 8.** Subgroup performance of the XGBoost model on the independent test set (n=5,311), stratified by sex and age group. Metrics are computed at the F2-maximising operating threshold (0.080), except AUC-ROC and its confidence interval, which are threshold-independent. Confidence intervals are 95% percentile bootstrap intervals (B=500 for subgroups), resampling cases and controls separately. The highlighted row identifies older adults as a critical equity concern.

| **Subgroup** | **N** | **Prev** | **AUC-ROC** | **95% CI** | **Sens** | **Spec** | **PPV** | **NPV** | **FPR** | **FNR** |
| --- | --- | --- | --- | --- | --- | --- | --- | --- | --- | --- |
| ***By sex*** |  | | | | | | | | | |
| Female | 2,765 | 14.6% | 0.783 | 0.760–0.804 | 0.783 | 0.603 | 0.253 | 0.942 | 0.397 | 0.217 |
| Male | 2,545 | 12.6% | 0.818 | 0.796–0.839 | 0.841 | 0.653 | 0.259 | 0.966 | 0.347 | 0.159 |
| ***By age group*** |  | | | | | | | | | |
| 10–17 | 715 | 18.0% | 0.803 | 0.755–0.844 | 0.814 | 0.609 | 0.314 | 0.937 | 0.391 | 0.186 |
| 18–24 | 1,313 | 11.4% | 0.817 | 0.785–0.849 | 0.720 | 0.714 | 0.245 | 0.952 | 0.286 | 0.280 |
| 25–34 | 1,165 | 12.9% | 0.800 | 0.764–0.832 | 0.840 | 0.604 | 0.239 | 0.962 | 0.396 | 0.160 |
| 35–44 | 828 | 14.7% | 0.809 | 0.769–0.846 | 0.877 | 0.567 | 0.259 | 0.964 | 0.433 | 0.123 |
| 45–54 | 621 | 15.0% | 0.774 | 0.728–0.819 | 0.839 | 0.570 | 0.256 | 0.953 | 0.430 | 0.161 |
| 55–64 | 338 | 13.0% | 0.751 | 0.673–0.821 | 0.750 | 0.605 | 0.221 | 0.942 | 0.395 | 0.250 |
| ≥65 | 286 | 8.7% | 0.722 | 0.617–0.822 | 0.680 | 0.716 | 0.187 | 0.959 | 0.284 | 0.320 |
| Unknown | 45 | 26.7% | 0.793 | 0.655–0.914 | 1.000 | 0.394 | 0.375 | 1.000 | 0.606 | 0.000 |

*Interval width varies markedly with the number of positive cases, from 0.043 in the largest strata to 0.205 in adults aged ≥65 years and 0.259 in the "Unknown" stratum, which comprises 45 attendances with unrecorded age and should not be interpreted. Differences in discrimination between age bands are not statistically distinguishable; the equity concern in older adults rests on sensitivity at the operating point rather than on discrimination.*

**Table 9.** Group-aware 5-fold cross-validation results using country as the grouping variable (GroupKFold). Each fold validates on one or more entire countries never seen during training, simulating deployment to a new national context. The highlighted row (Fold 1: Sweden) shows near-chance AUC-PR (0.069), attributable to 96.9% of Swedish records having Unknown outcome recoded as history-negative under the Third Way strategy, which compresses the validation signal. Excluding Sweden yields substantially improved and more homogeneous AUC-PR (0.294 ± 0.073), with essentially unchanged AUC-ROC (0.652 ± 0.027). The gap between test-set AUC-ROC (0.800) and cross-validation mean (0.658) is consistent with the implicit geographic encoding captured by the model.

| **Fold** | **N (train)** | **N (val)** | **Validation countries** | **Prevalence** | **AUC-ROC** | **AUC-PR** |
| --- | --- | --- | --- | --- | --- | --- |
| 1 | 17,597 | 8,950 | Sweden | 3.0% | 0.682 | 0.069 |
| 2 | 21,189 | 5,358 | Turkey | 11.9% | 0.656 | 0.204 |
| 3 | 21,538 | 5,009 | Latvia | 19.0% | 0.638 | 0.270 |
| 4 | 22,892 | 3,655 | Austria, Spain, Italy, Luxembourg | 22.4% | 0.689 | 0.333 |
| 5 | 22,972 | 3,575 | Cyprus, Czech Republic, Germany, Hungary, Malta, Poland, Romania | 26.4% | 0.626 | 0.371 |
| **Mean ± SD (all 5 folds)** | | | | | **0.658 ± 0.027** | **0.249 ± 0.119** |
| **Mean ± SD (excl. Sweden)** | | | | | **0.652 ± 0.027** | **0.294 ± 0.073** |

**Table 10.** Probabilistic bias analysis: model performance under alternative assignment of records coded 9. Records with an uncoded outcome were assigned to positive status with country-specific probabilities derived from the positivity rate observed among coded records in the same country, scaled by the factor shown. The 0% scenario corresponds to the primary analysis. The final column reports the discrimination achieved by recording country alone as a predictor of the simulated label, and quantifies the extent to which each scenario makes the outcome a function of nationality.

| **Scenario** | **Prevalence** | **AUC-ROC** | **AUC-PR** | **AUC-ROC, country alone** |
| --- | --- | --- | --- | --- |
| 0% of country rate (primary analysis) | 13.7% | 0.800 | 0.380 | — |
| 25% of country rate | 26.8% | 0.642 | 0.380 | 0.549 |
| 50% of country rate | 40.3% | 0.664 | 0.516 | 0.645 |
| 100% of country rate | 67.2% | 0.865 | 0.915 | 0.878 |

*At the 100% scenario, recording country alone predicts the simulated outcome better (AUC-ROC 0.878) than the full 743-feature model does (0.865). Because clinical features recover recording country with 95.3% accuracy, discrimination measured against these simulated labels reflects geographic encoding rather than outcome misclassification. These scenarios are therefore reported as a demonstration that country-based probabilistic reassignment is not identifiable with these data, not as estimates of the impact of outcome misclassification.*
